# Chromatin remodeling enzyme Brg1 is required for mouse lens fiber cell terminal differentiation and its denucleation

**DOI:** 10.1186/1756-8935-3-21

**Published:** 2010-11-30

**Authors:** Shuying He, Melinda K Pirity, Wei-Lin Wang, Louise Wolf, Bharesh K Chauhan, Kveta Cveklova, Ernst R Tamm, Ruth Ashery-Padan, Daniel Metzger, Akira Nakai, Pierre Chambon, Jiri Zavadil, Ales Cvekl

**Affiliations:** 1 Department of Genetics, Albert Einstein College of Medicine, Bronx, NY 10461, USA; 2 Department of Ophthalmology and Visual Sciences, Albert Einstein College of Medicine, Bronx, NY 10461, USA; 3 Institute of Human Anatomy and Embryology, University of Regensburg, D-93053 Regensburg, Germany; 4 Department of Human Molecular Genetics and Biochemistry, Sackler School of Medicine Tel-Aviv University, 69978 Ramat Aviv, Tel Aviv, Israel; 5 Institut de Génétique et de Biologie Moléculaire et Céllulaire, Université de Strasbourg, 1 rue Laurent Fries, 67404 Illkirch-France; 6 Department of Biochemistry and Molecular Biology, Yamaguchi University School of Medicine, Minami-Kogushi 1-1-1, Ube 755-8505, Japan; 7 Department of Pathology, NYU Cancer Institute and NYU Center for Health Informatics and Bioinformatics, New York University Langone Medical Center, New York, NY 10016, USA

## Abstract

**Background:**

Brahma-related gene 1 (*Brg1*, also known as *Smarca4 *and *Snf2β*) encodes an adenosine-5'-triphosphate (ATP)-dependent catalytical subunit of the (switch/sucrose nonfermentable) (SWI/SNF) chromatin remodeling complexes. SWI/SNF complexes are recruited to chromatin through multiple mechanisms, including specific DNA-binding factors (for example, heat shock transcription factor 4 (Hsf4) and paired box gene 6 (Pax6)), chromatin structural proteins (for example, high-mobility group A1 (HMGA1)) and/or acetylated core histones. Previous studies have shown that a single amino acid substitution (K798R) in the Brg1 ATPase domain acts via a dominant-negative (dn) mechanism. Genetic studies have demonstrated that Brg1 is an essential gene for early (that is, prior implantation) mouse embryonic development. Brg1 also controls neural stem cell maintenance, terminal differentiation of multiple cell lineages and organs including the T-cells, glial cells and limbs.

**Results:**

To examine the roles of Brg1 in mouse lens development, a dnBrg1 transgenic construct was expressed using the lens-specific αA-crystallin promoter in postmitotic lens fiber cells. Morphological studies revealed abnormal lens fiber cell differentiation in transgenic lenses resulting in cataract. Electron microscopic studies showed abnormal lens suture formation and incomplete karyolysis (that is, denucleation) of lens fiber cells. To identify genes regulated by Brg1, RNA expression profiling was performed in embryonic day 15.5 (E15.5) wild-type and dnBrg1 transgenic lenses. In addition, comparisons between differentially expressed genes in dnBrg1 transgenic, Pax6 heterozygous and Hsf4 homozygous lenses identified multiple genes coregulated by Brg1, Hsf4 and Pax6. DNase IIβ, a key enzyme required for lens fiber cell denucleation, was found to be downregulated in each of the Pax6, Brg1 and Hsf4 model systems. Lens-specific deletion of Brg1 using conditional gene targeting demonstrated that Brg1 was required for lens fiber cell differentiation, for expression of DNase IIβ, for lens fiber cell denucleation and indirectly for retinal development.

**Conclusions:**

These studies demonstrate a cell-autonomous role for Brg1 in lens fiber cell terminal differentiation and identified DNase IIβ as a potential direct target of SWI/SNF complexes. Brg1 is directly or indirectly involved in processes that degrade lens fiber cell chromatin. The presence of nuclei and other organelles generates scattered light incompatible with the optical requirements for the lens.

## Background

Eukaryotic DNA is organized as chromatin in the nucleus. Chromatin is a copolymer of DNA, histone and nonhistone proteins and small noncoding RNA. During embryonic development, specific regions of the genome alter their chromatin organization [1]. Gene expression is regulated at the level of the chromatin structure of individual genes and/or loci in the context of the three-dimensional organization of chromatin inside the cell nucleus. Local chromatin structure affects multiple stages of transcription, including the accessibility of sequence-specific DNA-binding transcription factors to promoters, enhancers and other genomic regulatory regions. Two major modifications of local chromatin structure (that is, chromatin remodeling) include posttranslational modifications of histones and adenosine-5'-triphosphate (ATP)-dependent alteration of nucleosomes [2].

ATP-dependent chromatin remodeling refers to dynamic processes in which multiprotein switch/sucrose nonfermentable (SWI/SNF), ISWI (Imitation Switch) and nucleosome remodeling and deacetylase (NuRD) complexes use nucleosomes as substrates and change positions of individual histone octamers and/or change the topology of DNA that is wrapped around the individual nucleosome particles [3]. Mammalian SWI/SNF complexes, SWI/SNF-A and SWI/SNF-B/polybromo-associated Brg1-associated factor (PBAF), are composed of a catalytical and several additional regulatory subunits, Brg1-associated factors (BAFs). Brg1 (Smarca4/Snf2β) and Brahma (Brm; Smarca2/Snf2α) are structurally similar chromatin remodeling ATP-dependent helicases that play distinct roles during embryonic development [4]. Brahma-related gene 1 (*Brg1*, also known as *Smarca4 *and *Snf2β*) is essential for early mammalian development as mutated embryos die during the preimplanation phase [5]. In contrast, loss of function of Brm leads to increased cellular proliferation in adult mouse tissues [6]. To study Brg1 function during organogenesis, conditional gene targeting of Brg1 was performed in T-cells [7], embryonic ectoderm/keratinocytes [8], hematopoietic/endothelial cells [9] and neural stem cells [10]. These studies found a wide range of cell autonomous defects, including the control of T-cell proliferation and survival [7], terminal differentiation of keratinocytes [8], differentiation and apoptosis of primitive erythrocytes [9] and neural stem maintenance and gliogenesis [10]. Mammalian SWI/SNF complexes participate in DNA double-strand break repair as they bind to the phosphorylated H2A histone family, member X (H2AX), histone variant, and promote its phosphorylation [11]. Recent studies have also established specific roles of Brg1 in DNA replication [12]. Additional insights into the role of Brg1 in muscle [13,14], mammary epithelium [15], smooth muscle [16,17] and myeloid [18] differentiation have been generated through the studies of a specific point mutation (K798R) in the ATP-binding domain of Brg1 that act via a dominant-negative (dn) mechanism [19]. In the eye, studies using zebrafish showed that Brg1 plays specific roles in lens and retinal development [20-22]. Eye differentiation defects found in zebrafish mutation *young *(*yng*) were linked to the presence of an Y390X mutation in the *Brg1*/*Smarca4 *gene on chromosome 3 [20,21]. Nevertheless, the existence of two *Brg1*-homologous genes, located on chromosomes 3 and 6 of the duplicated zebrafish genome, requires additional experimentation to clarify the roles of Brg1 enzymes in vertebrate eye development.

Central to understanding chromatin remodeling in embryonic development is to identify those genes that are regulated by specific chromatin remodeling systems and to elucidate the molecular mechanisms that recruit the remodelers to specific regions of chromatin. The molecular mechanisms of chromatin remodeling mediated by SWI/SNF complexes were probed using a combination of biochemical and genetic experiments. These experiments mostly examined the function of Brg1 as this enzyme alone can remodel nucleosomes [23,24]. Genes regulated by SWI/SNF complexes in vertebrate systems were identified using candidate gene approaches [15,25,26] and RNA expression profiling [14,22,27]. The SWI/SNF complexes are recruited to DNA by at least four different mechanisms. Several lineage-specific DNA-binding transcription factors, including cAMP response element-binding factor (CREB), Hsf4, microphthalmia-associated transcription factor (Mitf), Pax6 and T-box transcription factor 2 (Tbx2), were shown to associate with Brg1 using various *in vitro *protein interaction and whole cell extract coimmunoprecipitation assays [28-30]. Other transcription factors associate with Brg1-associated factor (BAF) subunits, that is, BAF60c interacts with retinoic acid receptor (RAR) and retinoid X receptor (RXR) heterodimers [31]. Brg1 contains a 110-amino-acid-long bromodomain that recognizes acetylated lysines in core histones [32]. Brg1 also interacts with chromosomal architectural proteins such as high mobility group A1 (HMGA1) [33]. Thus, CREB transcription factor, Hsf4 and Pax6 (see above) can potentially regulate lens development via recruitment of Brg1-containing SWI/SNF complexes [34,35].

Embryonic lens development is an excellent system to study both individual cell lineage formation and terminal differentiation. Lens lineage originates from the preplacodal region that is established around the anterior neural plate of the vertebrate embryo [36,37]. The lens placode, a thickened surface ectoderm, is the first morphologically distinct structure composed of lens progenitor cells. Invagination of the lens placode generates the lens vesicle, a polarized structure composed of lens precursor cells. The posterior cells of the lens vesicle exit the cell cycle and undergo terminal differentiation to generate primary lens fibers. The primary lens fibers are highly elongated cells filling the bulk of space of the original lens vesicle. The anterior cells of the lens vesicle subsequently differentiate into the anterior lens epithelium [38]. The lens grows through the entire lifespan as a result of epithelial cell division and migration toward the lens equator. When the epithelial cells reach the equator, they undergo terminal differentiation as secondary lens fibers. The hallmark of lens fiber cell differentiation is the expression of lens-preferred genes, the crystallins [34], and synchronized degradation of all subcellular organelles [39]. Lens fiber cell denucleation (karyolysis) is a final stage of this process that destroys the lens chromatin and/or epigenome. Aside from the active role of the acid DNase IIβ in this process, very little is known about molecular pathways that regulate lens fiber cell denucleation [39].

To investigate the role of Brg1 in lens fiber cell differentiation, we expressed a dn mutant of Brg1 using the lens-specific αA-crystallin promoter in postmitotic lens fibers. We examined lens growth and differentiation, focusing on the potential defects in the lens fiber cell denucleation process. Next, we identified differentially expressed genes in this system and compared these genes with genes regulated by Hsf4 and Pax6, two lineage-specific DNA-binding transcription factors shown to associate with SWI/SNF complexes through the Brg1 subunits [29,30]. The role of Brg1 during embryonic lens development was examined by conditional *Brg1 *gene inactivation in mouse embryos.

## Results

### Expression of Brg1 in mouse embryonic eye

**Figure 1:**
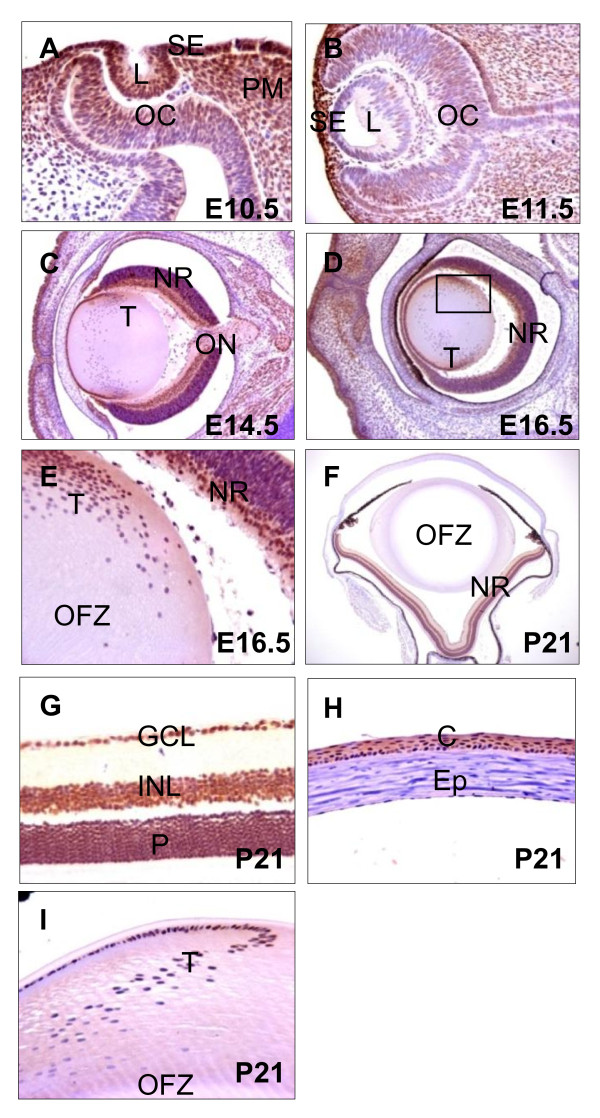
**Brahma-related gene 1 (*Brg1*) expression profile during mouse ocular development**. **(A-I) **Sagittal sections were immunostained with antibody recognizing Brg1 (brown) and counterstained lightly with hematoxylin (purple) at embryonic days E10.5 **(A)**, E11.5 **(B)**, E14.5 **(C) **and E16.5 **(D and E)**, as well as postnatal day P21 **(F-I)**. Higher-magnification areas stained with the Brg1 antibody indicated in **(D) **are shown in **(E)**. **(G-I) **Brg1 at different ocular regions of **(F)**. C: cornea, Ep: cornea epithelium, GCL: ganglion cell layer, INL: inner nuclear layer, L: lens, NR: neural retina, OC: optic cup, ON: optic nerve, P: photoreceptors, PM: periocular mesenchyme, SE: surface ectoderm, T: transition zone. Magnification: **(A)**, ×460; **(B and C)**, ×320; **(D)**, ×250; **(E)**, ×400; **(F)**, ×60; and **(G-I)**, ×320.

Previous studies using *in situ *hybridization in mouse showed that Brg1 is ubiquitously expressed and accumulates in differentiating cells in the nervous system, including brain, spinal cord and retina [40]. To explain the Brg1 loss-of-function studies performed here, we first determined the endogenous expression pattern of Brg1 during mouse eye embryonic development (embryonic days E10.5 to E16.5) and in 3-week-old (postnatal day 21, P21) mouse eye using immunohistochemistry as shown in Figure 1. Brg1 was detected at embryonic day 10.5 (E10.5) in the surface ectoderm, in the invaginating lens placode, in the periocular mesenchyme and in the anterior part of the optic cup, the prospective neuroretina (Figure 1A). By day E11.5, Brg1 staining was maintained in the surface ectoderm and the periocular mesenchyme, with speckled localization in the proliferating lens epithelial cells and in the anterior region of the optic cup (Figure 1B). In the differentiating lens (day E14.5), Brg1 was expressed in the lens epithelium and in primary lens fibers (Figure 1C). In addition, Brg1 was expressed predominantly in the surface ectoderm-derived tissues and in the neural retina. At day E16.5, Brg1 was found in the cornea, the lens, the neural retina and the optic nerve (Figure 1D). In the developing lens, the Brg1 proteins exhibited nuclear localization in the proliferating lens epithelium and the differentiating lens fiber cells that still retained their nuclei at the lens transitional zone (Figure 1E). At day P21 (Figures 1F-1I), strong Brg1 staining was detected in the photoreceptors (P), ganglion cell layers (GCL) and the inner nuclear layer (INL) of the retina (Figure 1G). In the lens, Brg1 expression was restricted to the single-layered lens epithelium and within the nuclei of fiber cells at the transitional zone (Figure 1I). In addition, a number of Brg1-positive cells were also visible in the corneal epithelium (Figure 1H). These data show that Brg1 is expressed both in lens progenitor cells and in early and late differentiating lens fiber cells.

### Expression of dominant-negative Brg1 (dnBrg1) in transgenic lens disrupts lens fiber cell differentiation and induces cataract formation

**Figure 2:**
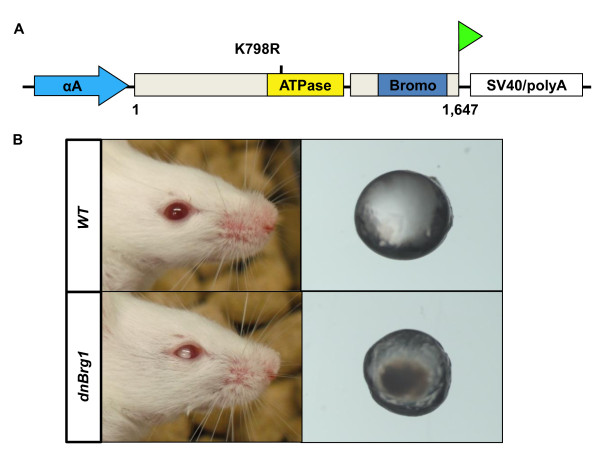
**Generation and initial evaluation of the dnBrg1 transgenic mice**. **(A) **Schematic of the transgenic construct. αA-crystallin promoter fragment (-366 to + 46, Cryaa; blue arrow) was used to express mutated Brg1 (K798R) with a C-terminal FLAG tag (green triangle). ATPase domain (yellow) and bromodomain (blue) are shown. **(B) **Two-month-old wild-type (WT) and transgenic mice. Note the cataract formation in the transgenic mouse and in isolated lens.

To address the function of Brg1 in mammalian lens development, we generated transgenic mice in which dnBrg1, tagged with C-terminal FLAG, was expressed using the tissue-specific αA-crystallin promoter (Figure 2A). This promoter fragment (-366 to + 46) drives heterologous gene expression from day E11.5 in postmitotic lens fiber cells [30,41,42]. The dnBrg1 contains a point mutation in the ATP binding motif at lysine residue 798 (Figure 2A), which is required for ATP-binding and helicase activity [19]. This mutation generated a dysfunctional protein that exerted dominant-negative effects versus wild-type Brg1 on transcription in yeast [19] and in cultured mammalian cell lines [13,14,17,18,43]. Three transgenic founders (B1 to B3) were initially obtained, and they all displayed similar lens abnormalities: the microdissected transgenic lenses were smaller and opaque compared to the wild-type lenses (Figure 2B). Immunofluorescence analysis via FLAG antibody confirmed transgene expression in differentiating primary lens fibers cells (data not shown). The B3 heterozygous transgenic founder was selected for subsequent studies, and the wild-type littermates were used as controls in each experiment.

### Analysis of lens fiber cell terminal differentiation in dnBrg1 transgenic mouse

**Figure 3:**
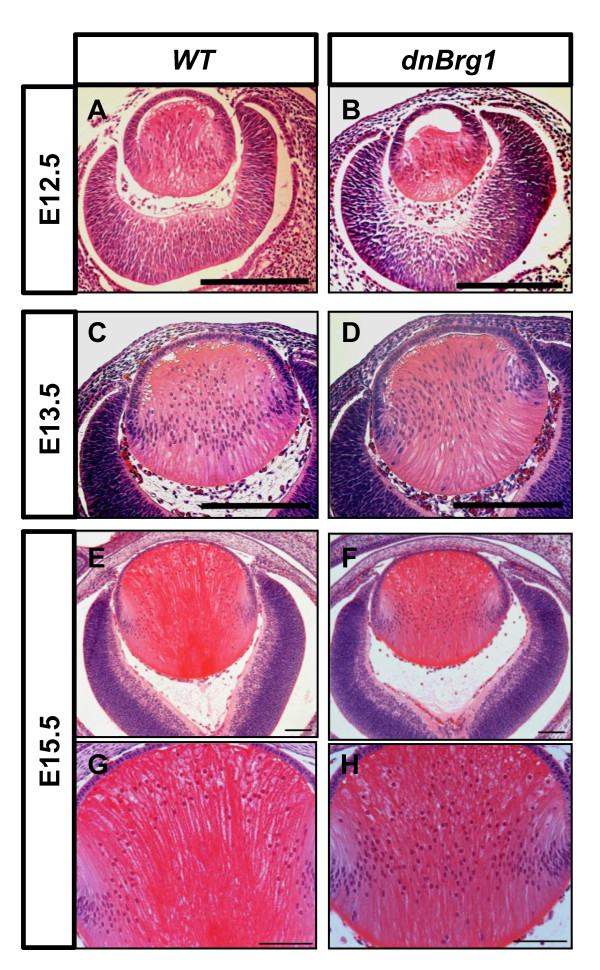
**Disruption of lens fiber cell differentiation in the dnBrg1 transgenic embryos**. Hematoxylin and eosin-stained lens midsections from wild-type **(A**, **C**, **E and G) **and dnBrg1 **(B**, **D**, **F and H) **embryos at different embryonic stages. Scale bar, 100 μm.

To evaluate the impact of transgenic dnBrg1 expression on lens development, we performed histological analyses of wild-type and dnBrg1 eyes at different developmental and postnatal stages (days E12.5 to P90). No changes were found at the onset of primary lens fiber cell differentiation at day E11.5 (data not shown). At day E12.5, wild-type lens primary fiber cells almost reached the lens epithelium in wild-type lens (Figure 3A). In contrast, transgenic primary lens fibers exhibited delayed cell elongation (Figure 3B). At day E13.5, both wild-type and dnBrg1 primary lens fiber cells filled the lumen of the lens vesicle (Figures 3C and 3D). However, in transgenic lenses, the nuclei of primary lens fiber cells were located more anteriorly compared to the wild-type lenses. In addition, transgenic lens fiber cells showed increased convex curvature, an indirect indication of abnormal fiber-to-fiber cell contacts. At day E15.5, normal lenses established the characteristic "bow" pattern of their nuclei (Figures 3E and 3G). In transgenic lenses, a large number of primary lens fiber cell nuclei were found scattered across the lens equator, which might indicate a delay of lens terminal differentiation in the transgenic embryos (compare Figures 3E and 3G with Figures 3F and 3H).

**Figure 4:**
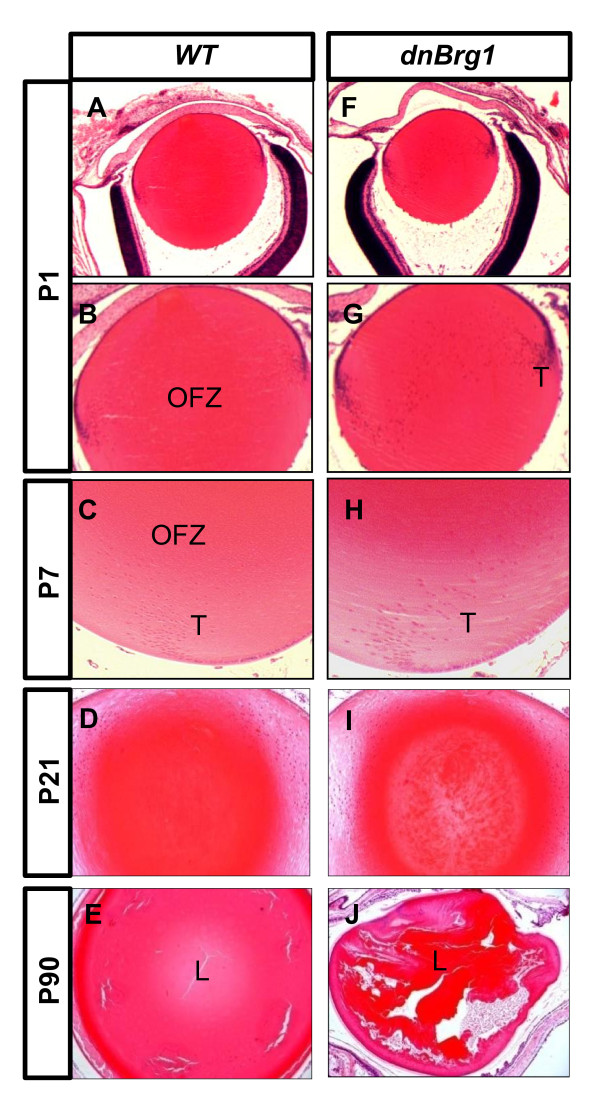
**Disrupted lens fiber cell denucleation and protein aggregation in postnatal dominant-negative (dn) Brg1 eyes**. Histological lens midsections were obtained from wild-type **(A-E) **and transgenic mice **(F-J) **from postnatal stages (days P1, P7, P21 and P90). Wild-type lenses gradually develop organelle-free zones (OFZs) from the center of the lens, but dnBrg1 transgenic lens fiber cell nuclei remain in the differentiated lens fiber cells **(F and G) **and are particularly evident at the lens transitional zones **(G and H)**. Lens protein aggregation was evident in day P21 **(I) **and day P90 **(J) **transgenic animals. Grossly distorted lens structure **(J) **was observed in the Brg1DN ocular system at day P90. L, lens; T, lens transitional zone.

In the postnatal dnBrg1 transgenic mice, lens abnormalities became more prominent and culminated with severe deterioration of the lens structure (Figure 4). In wild-type mouse, an organelle-free zone (OFZ) was established in the center of the lens, with the cortical lens fiber cells containing degrading nuclei (Figures 4A and 4B). In contrast, the dnBrg1 P1 lenses were smaller, with broader transitional zones and more fiber cell nuclei presented across the lens (Figures 4F and 4G). At day P7, a number of dnBrg1 lens fiber cells nuclei expanded from the transitional zone toward the center of the lens (Figure 4H). In contrast, in the transitional zone of the wild-type lens, fiber nuclei could hardly be discerned, as they were gradually undergoing denucleation (Figure 4C). In day P21 lenses, cataracts were formed in transgenic dnBrg1 lenses (Figure 4I), consistent with the external evidence of lens opacification (see Figure 2B). Finally, at day P90, the transgenic lenses showed disrupted internal microstructure characterized by distorted external shape and the presence of multiple large vacuoles (compare Figures 4E and 4J).

**Figure 5:**
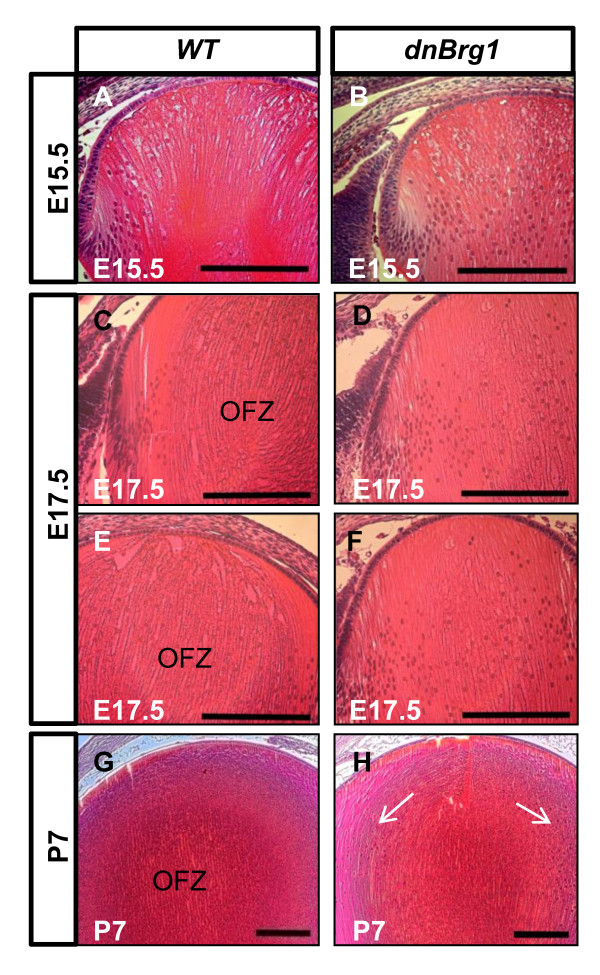
**Disruption of fiber cell nuclei degradation in embryonic and postnatal dnBrg1 transgenic lenses**. Lens midsections from both wild-type and Brg1DN mice from embryonic day 15. 5 **(A and B)**, embryonic day 17.5 **(C-F) **and postnatal day P7 **(G-H) **were stained with hematoxylin and eosin. Failure of fiber cell nuclei degradation (**H**, arrows) and organelle-free zone formation was observed in the transgenic mice from embryonic stage E17.5 and persisted in postnatal stages. Scale bar, 100 μm.

Lens fiber cell denucleation plays a major role in these cells' terminal differentiation. From days E13.5 to P7, various abnormalities associated with the position, number and morphology of nuclei are shown in Figures 3 and 4. To further address these issues, we examined lens fiber cell nuclear degradation and formation of the OFZ from the days E15.5 to P7 stages (Figure 5). The OFZ was formed in the wild-type lens of day E17.5 embryos (Figures 5C and 5E). In contrast, transgenic dnBrg1 fiber cell nuclei were retained and predominantly localized between the anterior lens epithelium and equatorial area (Figures 5D and 5F). At postnatal stage P7, the transgenic lens exhibited an expanded transitional zone at the expense of a smaller presumptive OFZ (compare Figures 5G and 5H).

**Figure 6:**
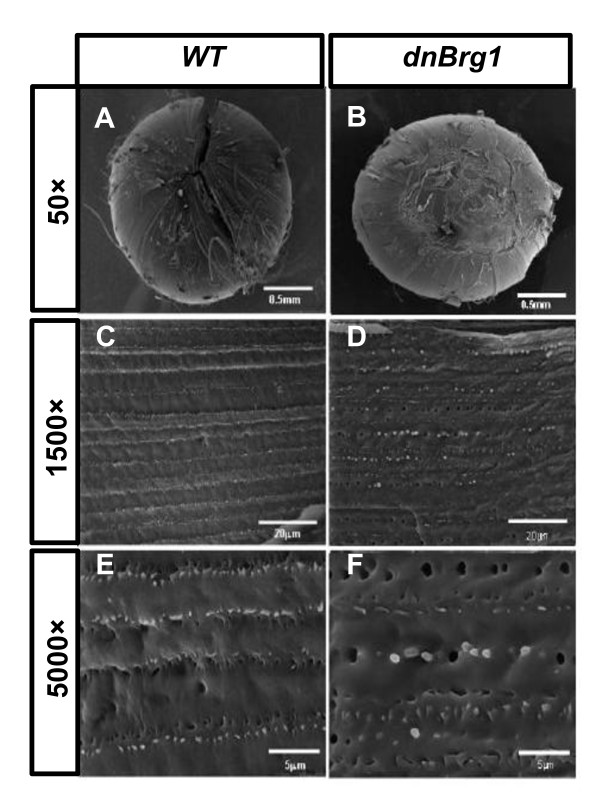
**Aberrant lens fiber cell organization in the dnBrg1 transgenic mouse**. Absence of "Y" suture formation (compare **A **and **B**) and ball-and-socket structure (compare **C **and **E **with **D **and **F**) revealed by scanning electron microscopy (SEM) suggested abnormal fiber cell differentiation in the transgenic mouse (age 3 mo). Altered fiber cell widths as well as a disturbed fiber cell organization pattern are shown at higher magnifications (**C-F**). Scale bars, 0.5 mm (**A-B**); 20 μm (**C-D**); 5 μm (**E-F**).

On the basis of the lens fiber cell morphological abnormalities observed in the transgenic mice, we reasoned that the dnBrg1 expression could affect lens fiber cell ultrastructure and contacts between individual lens fiber cells. Scanning electron microscopy (SEM) analysis of 6-month-old wild-type mouse lens revealed normal overlapped fiber cell layers forming three equally long suture branches as a "Y" pattern at the lens pole (Figure 6A), which is a characteristic of mice, rats, guinea pigs, cats, dogs and cows lenses [44]. The lens posterior pole demonstrated normal fiber end curvature and suture plane formation. The midcortical growth shells displayed uniform fiber cell mass and ordered arrangement of the ball-and-socket structure. In contrast, no typical "Y" suture pattern was found in the dnBrg1 lens (Figure 6B). The lens pole was fractured as the ends of most surface lens fibers failed to abut and overlap to form the three spherical suture branches. Consequently, the abnormal end-to-end arrangement of the lens fibers in the growing shells disturbed the formation of a smooth spherical lens surface (see Figures 6C-6F). In addition, the uniform thickness of the lens fibers and the organized suture pattern were absent in the transgenic lens [44]. The uneven width of the lens fibers was found in the transgenic mice, and the fiber cell "ball-and-socket" alignment pattern was perturbed (compare Figures 6E and 6F).

**Figure 7:**
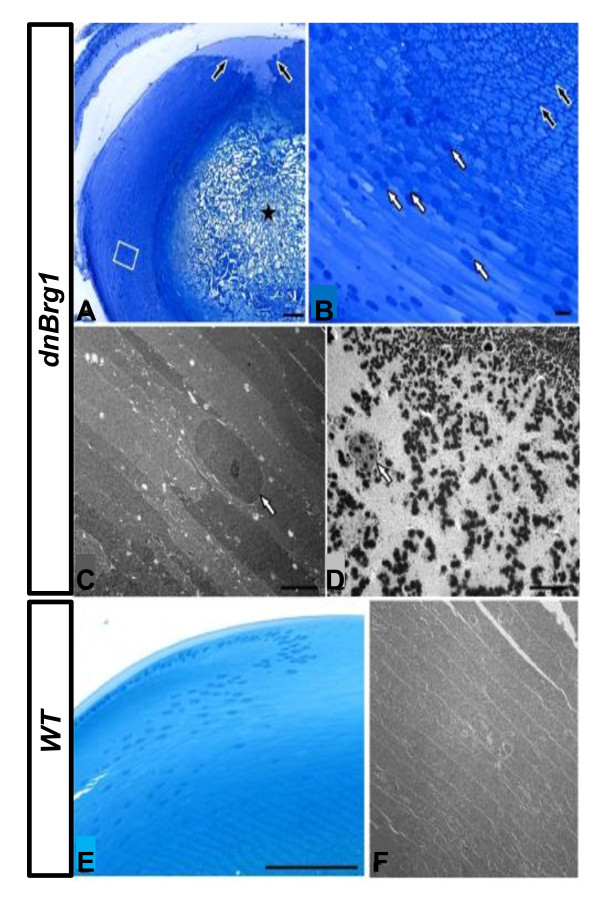
**Fine structure analysis of lens fiber cell abnormalities in the dnBrg1 transgenic lens**. Semithin sections **(A, B, and E) **and transmission electron microscopy **(C, D, and F) **of dnBrg1 transgenic lenses **(A-D) **and that of wild-type littermates **(E and F)**. **(A) **In lenses of dnBrg1 transgenic animals, the fibers in the core of the lens are completely disrupted and replaced by amorphous material (asterisk). At the posterior pole of the lens, fibers do not contact each other (black arrows). The boxed area is shown at higher magnification in **(B)**. **(B) **Lens fibers next to the central amorphous zone contain intensely labeled granular material close to their cell border, as well as some nuclei (black arrows). Nuclei are more frequent in more peripheral lens fibers (white arrows). **(C) **Nuclei (white arrow) of peripheral lens fibers show a homogeneous structure containing nucleoli. **(D) **Lens fibers that are localized more closely to the core contain numerous electron-dense granules with an average diameter of about 500 nm. Nuclei (white arrow) contain electron-dense granules of a size comparable to those seen in the peripheral cytoplasm, as well as numerous fine electron-dense particles that are considerably smaller. **(E and F) **Fibers of wild-type lenses show an overall ordered organization. Note that nuclei are only present in and next to the bow region. Scale bars, 100 μm **(A and E)**; 10 μm **(B)**; 5 μm **(C, D and F)**.

We next used analysis of 1-μm semithin sections followed by transmission electron microscopy (TEM) to further characterize lens fiber cell abnormalities in the dnBrg1 transgenic lens (Figure 7). In lenses of dnBrg1 transgenic animals, the fibers in the core of the lens were completely disrupted and replaced by amorphous material (Figure 7A). Lens fibers next to this amorphous zone appeared to be structurally intact, which contained intensely labeled granular material close to their cell border, as well as some nuclei (Figure 7B). Nuclei were more frequently observed in lens fibers that were arranged peripherally to those that showed intense staining at their cell border (Figure 7B). At the posterior pole of the lens, fibers did not contact each other (Figure 7B), which resulted in a fiber-free zone underneath the posterior lens capsule, an observation that correlated with the findings obtained by SEM. By TEM, nuclei were regularly observed in lens fibers close to the bow region. The nuclei showed a homogeneous structure and usually contained one nucleolus that was seen in cross sections (Figure 7C). The lens fibers that were localized more closely to the core, and which showed intense granular staining close to their cell border by light microscopy, contained numerous electron-dense granules with an average diameter of about 500 nm. Nuclei which were observed more rarely than in peripheral fibers contained electron-dense granules of a size comparable to those seen in the peripheral cytoplasm, as well as numerous fine electron-dense particles that were considerably smaller (Figure 7D). Fibers of wild-type lenses contained finely granular cytoplasm observed by TEM (Figure 7F) and showed an overall ordered organization by semithin light microscopy (Figure 7E). Nuclei were present only in and next to the bow region (Figures 7E and 7F).

To evaluate the expression of two key markers of lens fiber cell differentiation in wild-type and transgenic lenses, we used antibodies specific to αA-crystallin and major intrinsic protein of lens fibers (main intrinsic polypeptide (MIP), also known as aquaporin O and MIP26) to perform immunochemical staining. We found reduced expression of both lens structural proteins in the transgenic mice (Additional file 1). Next, to formally exclude the possibility that transgenic lens fibers reentered cell cycle, we performed bromodeoxyuridine (BrdU) incorporation assays and detected proliferating cells only in the lens epithelium (Additional file 2). From these morphological, microscopic and immunohistochemical studies, we concluded that lens-specific expression of dnBrg1 disrupted proper fiber cell organization and suture formation, which impaired the optical quality of transgenic lenses and contributed to progressive cataract formation.

### Identification of differentially expressed genes between dnBrg1 transgenic and wild-type lenses

**Figure 8:**
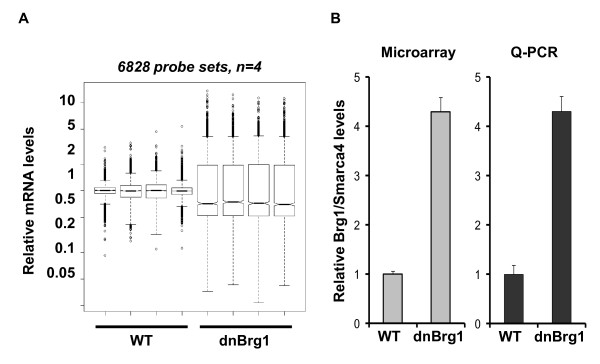
**Gene expression profiling in wild-type and dnBrg1 lenses (day E15.5)**. **(A) **Four biological triplicates (R1 through R4) of mouse lenses were analyzed by Affymetrix GeneChip arrays to identify a set of 6,828 individual genes significantly modulated between wild-type (WT) and Brg1DN lenses. Statistical filtering of array data was performed as described in Methods. **(B) **Differential expression of *Smarca4 *(*Brg1*) gene determined by the arrays and quantitative reverse transcriptase polymerase chain reaction (qRT-PCR).

We next performed RNA expression profiling using DNA microarrays to compare gene expression levels in wild-type and dnBrg1 transgenic embryonic (day E15.5) lenses. This time point was selected for the relative ease of dissecting lenses from mouse embryos with still relatively minor phenotypic changes (see Figure 3). Thus, the RNA expression data reflect changes in gene regulation approximately 72 hours after the onset of transgenic expression. Four sets of biological replicates were used for DNA microarray hybridization with Affymetrix Mouse Genome 430A 2.0 arrays (Affymetrix, Santa Clara, CA, USA). The statistical and bioinformatic analyses were performed as described in Methods. Initially, we identified 6,828 differentially expressed transcripts between the dnBrg1 transgenic and wild-type lenses from a total number of over 22,000 mouse genes represented on the array (Figure 8A). Among them, 3,208 were upregulated and 3,620 were downregulated transcripts, respectively. As expected, the microarray data revealed approximately fourfold upregulation of transcripts encoding Brg1, and this result was independently confirmed by quantitative real-time polymerase chain reaction (qRT-PCR) (Figure 8B). These results suggest that the mRNA encoding mutated and wild-type Brg1 mRNA were generated at a ratio of approximately 3:1 in day E15.5 transgenic lenses.

Several genes implicated in lens differentiation were found among the 6,828 differentially expressed transcripts. We selected 16 of these genes for secondary validation using qRT-PCR (Additional files 3 and 4). The analysis was carried out with cDNA prepared from independent pools of day E15.5 wild-type and dnBrg1 total lens RNA preparations as described in Methods. Internal controls (*B2m*, *Hprt and Sdha*) were included for data normalization [45]. These results confirmed upregulation of 12 genes (*Bfsp1*, *Cdkn1b/p27*, *Dnmt3a*, *Fgfr1*, *Gsn*, *Hif1a*, *Hod/Hop*, *Mab21l1*, *Prox1*, *Smarcd1*, *Smarce1 and Vim*) and downregulation of 3 genes (*Dnase2b*, *Jag1 *and *Pitpnm2*) in the dnBrg1 lenses (Additional file 3). Expression of Six3 showed no change on the arrays and minor downregulation by qRT-PCR (Additional file 3). In addition, we examined expression of Brm (Smarca2) in this system to address potential increase or decrease of expression of this ATPase [4]. We found a moderate upregulation of Smarca2 transcripts (1.92-fold for array data and 1.50-fold for qPCR data). Upregulation of *Smarcd1 *and *Smarce1*, two genes encoding noncatalytic subunits of SWI/SNF complexes, was also validated (Additional file 3). From these data, we concluded that approximately one third of the lens transcriptome was directly or indirectly affected by the transgenic overexpression of dnBrg1 with comparable numbers of up- and downregulated transcripts.

**Figure 9:**
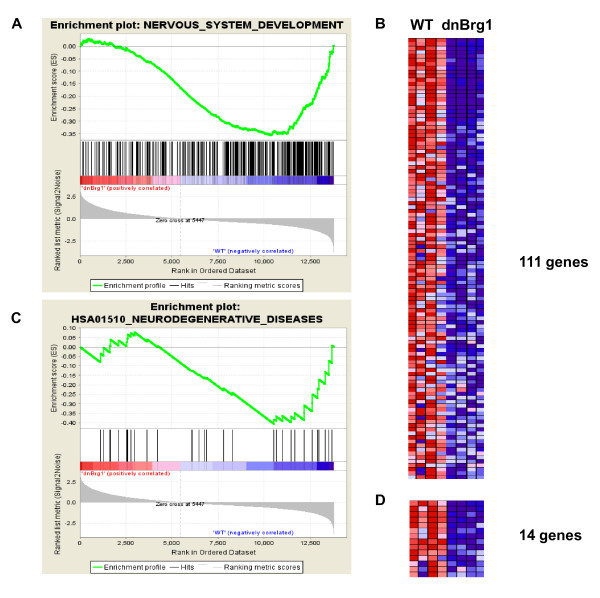
**Gene Set Enrichment Analysis (GSEA) analysis of two significantly enriched classes of genes: nervous system development and neurodegenerative diseases**. **(A) **Enrichment plot for a category nervous system development that includes 300 genes. **(B) **Normogram for 111 core-enriched genes classified in nervous system development. **(C) **Enrichment plot for a category of neurodegenerative diseases that includes 34 genes. **(D) **Normogram for 14 core enriched genes classified in neurodegenerative diseases. Individual genes are listed in Additional file 5. Increased expression (red); decreased expression (blue).

To further explore the dnBrg1-affected transcriptome remodeling, Gene Set Enrichment Analysis (GSEA) [46] was employed to identify significantly enriched molecular signatures disrupted in this transgenic model on the basis of normalized enrichment score (NES) ranking. Next, we assigned those groups into three categories: chromatin, lens biology and neuronal function, as shown in Additional file 5. The two most enriched signatures in the downregulated gene population (Figure 9) contained genes classified as "nervous system development" and "neurodegenerative diseases" with 37% (111 of 300) and 42% (14 of 33) respective signature enrichment, suggestive of disruption of Brg1-dependent programs associated with neural development as reported previously [10].

**Figure 10:**
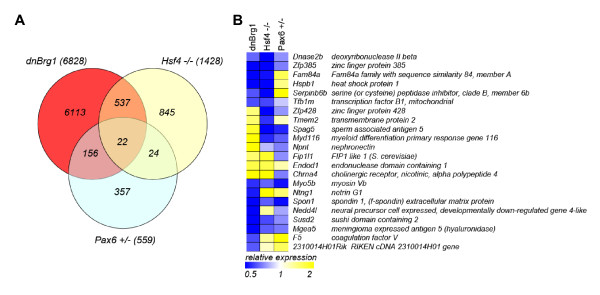
**Identification of lenses commonly regulated in *dnBrg1*, *Hsf4***^***-/- ***^**and *Pax6***^***+/-***^. **(A) **Venn diagram showing number of genes and/or transcripts regulated in *dnBrg1*, *Hsf4*^*-/- *^and *Pax6*^*+/- *^lenses. **(B) **A list of 22 genes regulated in all three systems. The genes were analyzed using the unsupervised hierarchical clustering described in Methods. Gene Expression Omnibus database accession numbers for dnBrg1, heat shock transcription factor 4 (Hsf4) and paired box gene 6 (Pax6) data are GSE22322, GSE22362 and GSE13244, respectively.

To identify individual genes regulated by Brg1 that explain lens fiber cell differentiation defects, we looked for genes that were deregulated in two functionally related systems: Pax6 and Hsf4. Pax6 regulates multiple stages of lens development [47,48]. In contrast, Hsf4 regulates lens fiber cell terminal differentiation [49]. As Pax6 [30] and Hsf4 [29] have been implicated as specific DNA-binding transcription factors that recruit Brg1 (and by inference SWI/SNF complexes) to specific regions of the genome, we further compared transcripts regulated in dnBrg1 transgenic lenses with the *Pax6*-heterozygous [45] and *Hsf4*-null lenses (see Methods). We found a total of 178 deregulated transcripts in both dnBrg1 and Pax6 heterozygous lenses (Figure 10A). In Hsf4-null lenses, we identified 1,428 differentially expressed transcripts. Among those, 559 transcripts were differentially expressed in both dnBrg1-transgenic and Hsf4-null lens models. Twenty-two differentially expressed genes were found in all three experiments, and they are listed in Figure 10B. This group included *Dnase2b*, a gene encoding acid nuclease DNase IIβ [39]. This enzyme is critical for lens fiber cell denucleation [39,50] and its downregulation in dnBrg1 transgenic lenses (Additional file 3) is consistent with lens fiber cell denucleation defects described above (see Figures 4 and 5).

Using the Database for Annotation, Visualization and Integrated Discovery (DAVID), we further classified those 178 genes that were commonly deregulated in both Pax6 heterozygous and dnBrg1 transgenic lenses (Figure 10A). A total of 130 genes (73%) were classified using the DAVID tool. Among these, there were 63 individual genes with partially redundant presence in multiple Gene Ontology (GO) categories. For example, the *Dnase2b *gene was found in GO Biological Process category "DNA metabolic process," GO Molecular Function category "nuclease activity," GO Cellular Compartment "vacuole" and Kyoto Encyclopedia of Genes and Genomes (KEGG) Pathway "lysozome" (Additional file 6). Among the top-ranking GO categories we identified were GO Molecular Function "nucleotide binding" (*n *= 20, *P *= 0.005), "DNA metabolic process" (*n *= 6, *P *= 0.043) and "DNA replication" (*n *= 4, *P *= 0.032). These results are in agreement with Brg1's serving as a global regulator of chromatin and chromatin-associated processes including DNA repair [11] and DNA replication [12].

### Targeted deletion of Brg1 causes multiple eye developmental abnormalities

**Figure 11:**
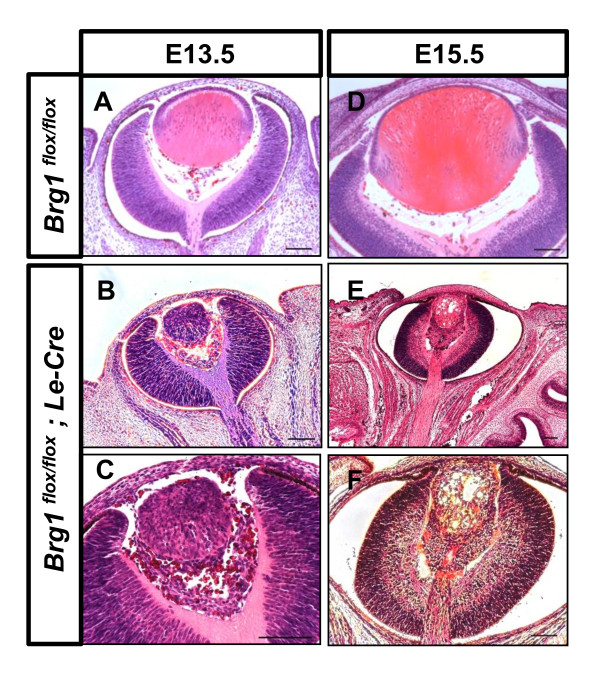
**Early deletion of Brg1 compromise ocular morphogenesis in the conditional knockout (cKO) mutant embryos**. Lens midsections were obtained from *Brg1*^*flox/flox *^and *Brg1*^*flox/flox*^; *Le-Cre *(mutant) mice from embryonic day 13.5 **(A-C) **and embryonic day 15.5 **(D-F)**. The examined Brg1 mutant embryos still underwent lens induction but revealed disrupted lens specification as well as an abnormal neural retina during embryonic development. Scale bar, 100 μm.

To further examine the role of Brg1 in the regulation of lens morphogenesis, we performed conditional knockout (cKO) of Brg1 by crossing *Brg1*^*flox/flox *^[7] mice with *Le-Cre*-transgenic mice [47]. The *Le-Cre *mouse is used to inactivate genes in the embryonic head surface ectoderm cells that give rise to the lens [47,51,52]. The onset of Le-cre activity was previously reported to commence around day E9.0 [47], corresponding with the activity of the Pax6 ectodermal enhancer (EE) [53]. The *Brg1*^*flox/flox *^mice and the *Le-Cre *mice did not show any phenotypic differences in comparison with the wild type (Figure 11 and data not shown). Therefore, we used the *Brg1*^*flox/flox *^mice as controls in our experiments. Following this Cre-lox-mediated deletion strategy, *Brg1*^*flox/flox*^, *Le-Cre *and *Brg1*^*flox/+ *^*Le-Cre *conditional knockout mice were obtained in the expected Mendelian ratios. The *Brg1*^*flox/+ *^*Le-Cre *mice appeared normal in terms of lens gross morphology (data not shown). These findings suggest that one allele of *Brg1 *was sufficient for normal eye development. In contrast, the majority of *Brg1*^*flox/flox *^*Le-Cre *mice (referred to herein as *Brg1 *cKO mutants) showed severe microphthalmia (that is, reduced size of the eye) as visually identified after birth (Additional file 7). Analysis of 47 Brg1 cKO embryos revealed different severity of ocular abnormalities in lens, retina and other tissues in 70% of the conditional knockout mutants (Table 1). In addition, in 2 of 47 embryos, aphakia (that is, absence of lens) was found. Analysis of the efficiency of the conditional knockouts revealed that in many embryos, deletions of both *Brg1 *alleles were not complete (Additional file 8). These results suggest that conditional inactivation of *Brg1 *in the surface ectoderm triggers a cascade of ocular developmental abnormalities in both the anterior and posterior eye segments.

**Table 1:** A summary of gross morphology of the lens-specific Brg1 conditional knockout mice

	Total number of embryos	No obvious phenotype	Abnormal eye development	Aphakia
Brg1^flox/+ ^; Le-Cre	29	29	1	0
Brg1^flox/flox^; Le-Cre	47	14	31	2

**Figure 12:**
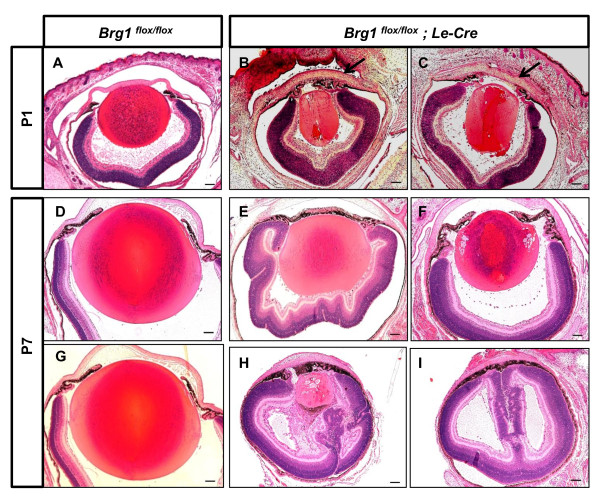
**Lens-specific Brg1 targeting gives rise to a variety of ocular deficiencies**. The cKO mutants were compared with *Brg1*^*flox/flox *^littermates at postnatal day 1 **(A-C) **and postnatal day 7 **(D-I)**. The mutant mice displayed ocular defects with various severities, including failure of cornea-eyelid separation (black arrows in **B **and **C**) or iris separation (**E**, **I **and **H**), aberrant lens and retina (**B**, **C**, **E**, **F**, and **H**) and even an absence of lens structure (**I**). Scale bar, 100 μm.

Morphological and histological analysis of ocular defects in Brg1 mutants in embryonic and postnatal eyes is shown in Figures 11 and 12, respectively. At day E13.5, the Brg1 mutant lenses were reduced in size compared to the controls (Figures 11A, 11B and 11C). A clear discrimination between the lens epithelium and the lens fiber cells is also missing. In some cases, no anterior and posterior chambers were generated as the lens epithelial layer remained attached to the surface ectoderm, and the mesenchymal cells between posterior lens and the optic nerve failed to degrade (Figures 11B and 11C). Reduced lens growth and abnormal lens fiber cell differentiation were also found at day E15.5 (Figures 11E and 11F). In the Brg1 cKO embryos, the neural retina was much thicker at both day E13.5 (Figures 11B and 11C) and day E15.5 (Figures 11E and 11F). In addition, abundant mesenchymal cells were presented in the vitreous region between the posterior of the lens and the neuroretina (Figures 11E and 11F). At day E15.5, the mutant lens fiber cells failed to elongate properly and vacuoles could be seen across the lens microstructure (Figures 11E and 11F). In the postnatal Brg1 mutants (P1 and P7 stages), reduced lens size and other abnormalities, including retinal misfolding, persisted (Figures 12B, 12C, 12E, 12F, and 12H). The most severe defect, aphakia (Figure 12I), was accompanied by an aberrant infolding of the retina. In some animals, the anterior segment abnormalities included failures in corneal differentiation, formation of the anterior chamber and cornea-eyelid separation (Figures 12B and 12C). In adults, the mutant eyelids were often closed (Additional file 7B). The conditional inactivation of *Brg1 *in the presumptive lens ectoderm shows that this enzyme is cell-autonomously required for lens fiber cell differentiation, indirectly for normal retinal and directly and/or indirectly for anterior segment development.

**Figure 13:**
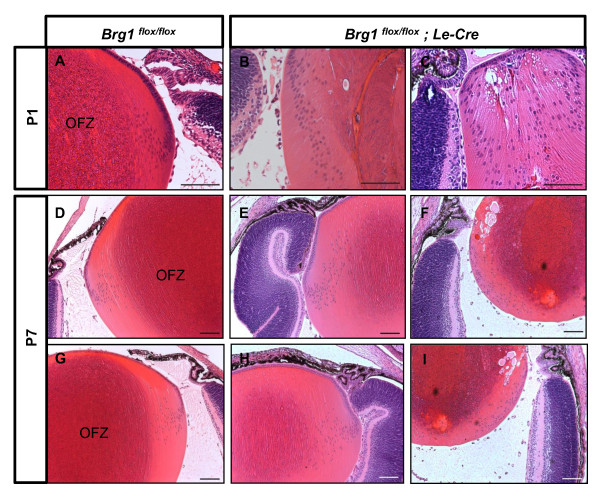
**Loss of function of Brg1 abrogates lens fiber cell denucleation**. Lens midsections were collected from both wild-type mice and littermates with lens-specific gene targeting of Brg1 from postnatal day 1 **(A-C) **and postnatal day 7 **(D-I) **for hematoxylin and eosin staining. OFZ, organelle-free zone. Scale bar, 100 μm.

**Figure 14:**
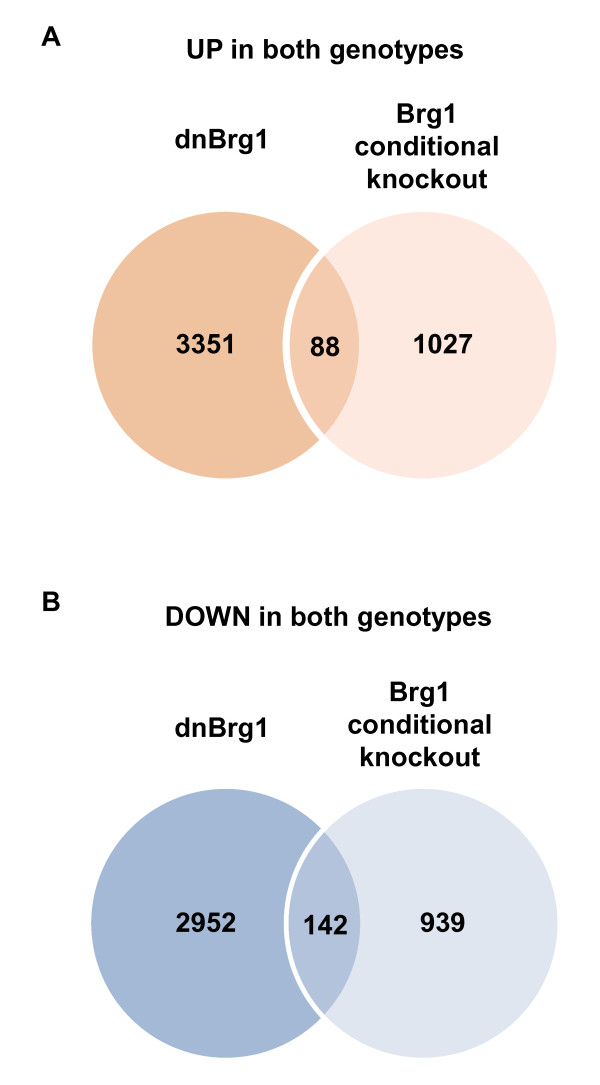
**Identification of commonly regulated genes in dnBrg1 lenses and Brg1 cKO eyes**. **(A) **Venn diagram showing numbers of upregulated genes and/or transcripts found in dnBrg1 and Brg1 cKO models. **(B) **Venn diagram showing numbers of downregulated genes and/or transcripts found in dnBrg1 and Brg1 cKO models. The genes and/or transcripts included in this analysis (6,533 in dnBrg1, and 2,196 in Brg1 cKO, respectively) were selected by two parameters: Pavlidis template matching (PTM), *P *< 0.05, and fold change > 1.5 as described in Methods [68].

Analysis of lens fiber cell denucleation in Brg1 mutants revealed retention of nuclei as well as abnormal shape of the lens fiber cells (Figure 13). Thus, in both dnBrg1 transgenic and in Brg1 cKO lenses, lens fiber cell karyolysis was disrupted. In addition, we found perturbed expression patterns of αA- and γ-crystallin proteins in Brg1 mutants (Additional file 9). Expression of Pax6 was detected in the lens epithelium of the Brg1 mutated lens (Additional file 9). To gain further insight into both models of Brg1 function, we performed transcriptome analysis in Brg1 mutants using whole eyeballs with three sets of biological replicates and the Affymetrix Mouse Genome 430A 2.0 arrays (Affymetrix, Santa Clara, CA, USA) as described in Methods. The initial analysis revealed disrupted expression of 2,196 genes, with 1,115 upregulated and 1,081 downregulated genes. Interestingly, only a minor fraction (230) of modulated genes (constituting 3.5% of dnBrg1- and 10.5% of Brg1 cKO-deregulated genes) showed similar behavior in these two loss-of-function model systems (Figure 14). These 230 genes are listed in Additional file 10. Nevertheless, expression of *Dnase2b *was reduced in both of these systems (Additional files 3 and 10). Collectively, our molecular profiling data establish specific roles of Brg1 in mouse embryonic lens fiber cell differentiation and their denucleation.

## Discussion

Using a combination of two complementary genetic approaches, the present studies demonstrate that Brg1 is required for mouse lens fiber cell differentiation. Lens-specific expression of the dnBrg1 perturbed lens fiber cell differentiation process at multiple levels and resulted in cataract formation. In these abnormal lens fibers, nuclei were not degraded, suggesting that Brg1 participates in normal lens fiber cell karyolysis. The advantage of this system is that function of Brg1 was disrupted only in postmitotic lens fiber cells; however, this system is unlikely to produce complete inactivation of Brg1 biological activity [52]. To address this problem, conditional inactivation of Brg1 using a MLR39 Cre line, active only in differentiating lens fibers, would be required [54]. In parallel, conditional inactivation of *Brg1 *in the surface ectoderm resulted in a range of lens and/or eye developmental abnormalities, including the retention of nuclei in lens fiber cells. Incomplete deletion of floxed Brg1 alleles by Le-cre followed by clonal selection of viable cells and/or prolonged stability of Brg1 proteins in lens cells precludes any definitive conclusions about the potential role of Brg1 in lens lineage formation and lens placode invagination. In the majority of mutants, lens vesicles were formed and differentiation of primary lens fibers was compromised. Absence of lens in the mutated eye was accompanied by an aberrant infolding of the retina. Similar defects were found in Pax6 embryos conditionally inactivated in the surface ectoderm [47]. Collectively, the present studies reveal an essential, novel role of Brg1 in lens fiber differentiation and denucleation. In addition, secondary defects in retinal formation suggest that Brg1 can play cell nonautonomous roles in retinal development that originate from aberrant lens morphogenesis as described elsewhere [47,52,55-58].

Although the use of two loss-of-function approaches to study the Brg1 function in lens development generated comparable results at the morphological and cellular levels, molecular studies using RNA expression profiling identified only a small number of commonly regulated genes (Figure 14). There are at least three factors that could contribute to these findings. First, loss of function of Brg1 from day E9.0, that is, prior to the morphological formation of lens pit and/or vesicle, should impair lens development more severely compared to the transgenic dnBrg1 system with later onset expression (from day ~ E11.5) in postmitotic lens fibers. Second, we could not isolate mutated lenses from comparable, that is, E15.5-day-old, embryos because of their structural fragility. Instead, we had to dissect eyeballs including mutated lenses and other affected tissues, and this tissue heterogeneity was reflected in the eyeball transcriptome. Third, the variability of the Brg1 cKO phenotypes (Table 1 and Figure 12) makes it difficult to microdissect lenses, even under ideal conditions, with abnormalities comparable to the dnBrg1 transgenic lenses.

Previous studies of Brg1 function in other cells and tissues established Brg1 as a specific regulator of cell proliferation, differentiation and survival [7-9,11,12]. The present studies in lens suggest that Brg1 plays a major role in lens fiber cell differentiation. Though Brg1 is highly expressed in the surface ectoderm that gives rise to the lens placode (Figures 1A and 1B), Brg1's role in the formation of lens lineage remains to be determined through detailed analysis of early stage (days E9-E10) embryos. Expression of Brg1 is reduced in differentiating primary and secondary lens fibers. Brg1's role in lens fiber cell differentiation is supported by ectopic expression of the dnBrg1 transgene in lens and by conditional inactivation of Brg1 in the presumptive lens ectoderm. Three transgenic mouse lines were established and generated similar lens-specific differentiation defects. Although the lens-specific knockout resulted in variable eye defects, in the majority of embryos, we detected rudimentary lens formation. This variability could originate from incomplete deletion of both *Brg1 *alleles, compensation via Brm/Smarca2 and/or via other mechanisms such as prolonged stability of the Brg1 proteins. Upregulation of Brm/Smarca2 was indeed found in the dnBrg1-transgenic model. A large number of transgenic lens studies utilizing the αA-crystallin promoter induced cell cycle reentry and/or apoptosis in the lens fiber cell compartment [41,42,59-62]. In the present study, no evidence for apoptosis (data not shown) and cell cycle reentry (see Additional file 2) in postmitotic dnBrg1 transgenic lens fibers was found.

Earlier studies identified αA- and αB-crystallins (among ~ 80 other genes) upregulated in the human adrenal carcinoma cell line SW-13, deficient in both Brg1 and Brm expression, in which Brg1 was reintroduced [27,33]. Here we show reduced expression and accumulation of αA-crystallin in dnBrg1-transgenic and Brg1-cKO lenses that is consistent with our earlier findings that abundant quantities of Brg1 are present within a 16-kb region of lens-specific chromatin of the mouse *Cryaa *locus [30]. On the basis of the data shown here and in our earlier studies [30], we conclude that αA-crystallin gene and/or locus is regulated directly by at least three DNA-binding transcription factors, Pax6, c-Maf and CREB, and by chromatin remodeling enzyme Brg1. We propose that Brg1, and, by inference, SWI/SNF complexes, are recruited to the *Cryaa *locus by Pax6 and possibly by other mechanisms, including the recognition of acetylated lysine residues by Brg1 bromodomain.

To identify genes downstream of Brg1 in lens, we performed RNA expression profiling studies in day E15.5 dnBrg1 transgenic lenses followed by comparative analyses of differentially expressed genes in Pax6 heterozygous and Hsf4 homozygous lenses. We reasoned that if lens lineage-specific DNA-binding transcription factors Pax6 and Hsf4 serve *in vivo *as recruiters of SWI/SNF complexes to specific regions of chromatin, we could find commonly regulated genes in these three model systems. Among those 6,828 transcripts regulated in dnBrg1 transgenic lenses, 715 (~ 10.5%) transcripts were also regulated in *Pax6*^*+/- *^and *Hsf4*^*-/- *^mutated lenses. Within the 559 differentially expressed genes in *Pax6*^*+/- *^lenses, 178 (~ 32%) transcripts were shared between these two systems. Similarly, within the 1,428 differentially expressed genes in *Hsf4*^*-/- *^mutated lenses, 559 (~ 39%) transcripts were commonly deregulated. Finally, 22 genes, including lens-preferred acid DNase IIβ endonuclease, were found to be dysregulated in all three mutated lenses. These results suggest that Brg1, Hsf4 and Pax6 exert their function through commonly regulated genes. In addition, the use of the DAVID and GSEA analysis tools for interpretation of genomewide expression profiles identified several functionally related groups of genes suggesting the presence of specific Brg1-dependent coregulated biological processes. Additional molecular studies using chromatin immunoprecipitation (ChIP) sequencing (ChIP-seq) and related methods are required to demonstrate co-localization of Brg1, Hsf4 and Pax6 proteins in lens chromatin at their target genes.

**Figure 15:**
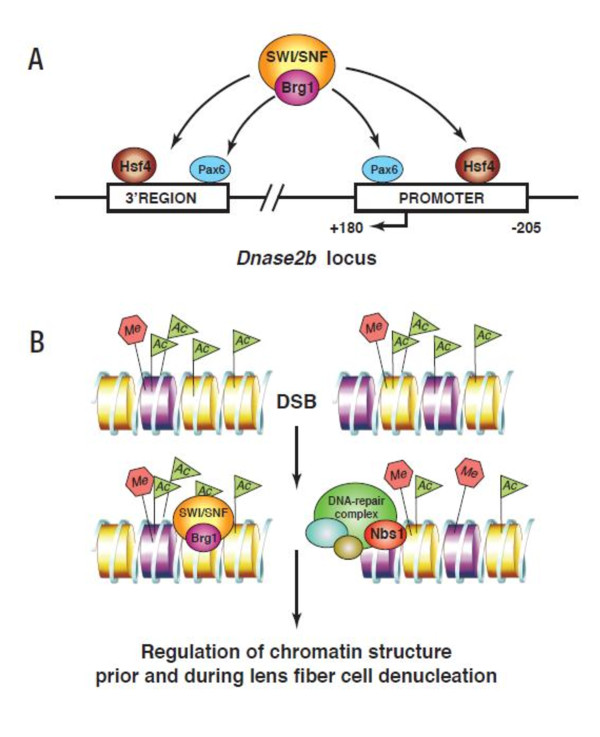
**Summary of two complementary models illustrating Brg1's role during lens fiber cell differentiation**. **(A) **A schematic of the *Dnase2b *locus including its evolutionarily conserved promoter region (-205 to + 180). Multiple Pax6- and Hsf4-binding sites were identified in the *DNase2b *promoter and 3'-downstream evolutionary conserved region. Hsf4 and Pax6 recruit (switch/sucrose nonfermentable) (SWI/SNF) complexes as described elsewhere [29,30]. **(B) **A schematic of a DNA double-strand break (DSB) accompanied by insertions of H2A histone family, member X (H2AX) histone variant (nucleosomes shown in purple). Both SWI/SNF (including Brg1) and DNA repair (including Nbs1) complexes are then recruited to the chromatin. Both complexes are thought to regulate chromatin structure prior to and during lens fiber cell denucleation. In mouse, Nbs1-deficient lenses show incomplete denucleation of lens fiber cells [71].

The most obvious defects in dnBrg1 transgenic lens fiber cell differentiation included the failure of lens fiber cells to degrade their nuclei, abnormal curvature and cell-to-cell contacts of lens fiber cells, and suture formations. All of these processes likely contributed to lens opacities found in postnatal transgenic lenses. The most interesting aspect of abnormal lens fibers was retention of their nuclei. These findings add a novel role for Brg1 to control denucleation and/or karyolysis as an important process of terminal differentiation. Downregulation of Dnase2b transcripts in dnBrg1 transgenic lenses as well as in conditionally deleted *Brg1 *lens can explain, at least partially, this phenotype. In wild-type lenses, DNase IIβ reaches its peak activity at day E17.5 [63], and this is followed by the establishment of an OFZ. In both the dnBrg1-transgenic and mutated Brg1 lenses, formation of an OFZ was not found (Figures 4 and 13). Initial analysis of the *Dnase2b *promoter and its surrounding regions identified multiple putative Pax6- and Hsf4-binding sites (Figure 15A), suggesting that these two transcription factors regulate lens-preferred expression of this enzyme via recruitment of SWI/SNF complexes. In addition to DNase IIβ, the present studies suggest a large number of differentially expressed genes that belong to GO Biological Process categories: DNA repair, establishment and/or maintenance of chromatin architecture, response to DNA damage stimulus and chromatin modification (see Additional file 11). Given the recent link between ubiquitin metabolism and lens fiber cell denucleation [64], we also identified differentially expressed genes that belong to the GO categories ubiquitin cycle, proteasome and ubiquitin-mediated proteolysis. These genes are excellent candidates for mechanistic studies of lens fiber cell denucleation. Finally, it is also possible that SWI/SNF complexes containing Brg1 participate in the process of lens fiber denucleation independently through controlling local chromatin structure in those regions that exhibit initial DNA damage. This is supported by our recent study that revealed formation of DNA double-stranded breaks repair through visualization of phosphorylated H2AX in the lens fiber cell compartment [41]. Both models of Brg1's role (and by inference the role of SWI/SNF complexes) in lens fiber cell denucleation (Figure 15) are not mutually exclusive. Ongoing experiments are aimed to test these hypotheses.

## Conclusions

Our results demonstrate that lens fiber cell terminal differentiation, including their denucleation (karyolysis), requires the ATP-dependent chromatin remodeling enzyme Brg1. Our data suggest that Brg1, together with two lens lineage transcription factors, Pax6 and Hsf4, is required for the transcriptional regulation of DNase IIβ, the key enzyme for lens fiber cell denucleation. In addition, the present data are consistent with our earlier findings suggesting that Brg1 regulates directly the expression of the αA-crystallin gene, the key structural protein of the mammalian lens. These results provide new molecular insights into the process of lens fiber terminal differentiation and open new research avenues to probe chromatin structure prior to and during lens fiber cell denucleation.

## Methods

### Antibodies

The primary antibodies used were Brg1, αA-crystallin, γ-crystallin, Pax6, MIP/MIP26/aquaporin 0 (Santa Cruz Biotechnology, Santa Cruz, CA, USA) and Flag M2 (Sigma-Aldrich, St. Louis, MO, USA). The secondary antibodies used were Alexa 568 goat anti-rabbit (Molecular Probes, Eugene, OR, USA) and biotin-conjugated secondary anti-rabbit (EO466; Dako, Carpintera, CA, USA).

### Generation of transgenic dnBrg1 mice

The plasmid pACP3 [59] was modified to insert *Eco*RV and *Mlu*I restriction sites into both 5'- and 3'-*Not*I sites that flank the αA-crystallin promoter/polylinker/simian virus 40 (SV40)/polyA sequences. A Brg1 cDNA containing the K798R mutation was cloned as a 5.2-kb *Cla*I-*Spe*I fragment obtained from pBluescript KS(+)-FLBrg1Mut [23] to generate pCryaa/dnBrg1 (see Figure 2A). A 7.2-kb *Eco*RV fragment was released from this plasmid and used to generate transgenic mice by injection into FVB/N fertilized oocytes. Transgene integration was confirmed by genomic PCR using tail DNA with the following primers: 5'-ATGGCTCCAGGGGAAGG-3' and 5'-CATTCCTTTCATCTGGTTG-3'. The cycling parameters were 94°C for 30 s, 55°C for 45 s and 72°C for 70 s for 35 cycles.

### Conditional inactivation of Brg1 in the presumptive lens ectoderm

*Brg1*^*flox/flox *^mice [7] were mated with *Le-Cre*-transgenic mice [47], and the progeny were crossed to generate litters containing homozygous floxed alleles and heterozygous for the Cre transgene. Mice genotyping was performed as described previously in the literature [7,47]. Animal husbandry and experiments were conducted in accordance with the approved protocol of the Albert Einstein College of Medicine Animal Institute Committee and the Association for Research in Vision and Ophthalmology Statement for the use of animals in ophthalmic and vision research. Noon of the day the vaginal plug was observed was considered to be day E0.5 of embryogenesis.

### Histological analysis

Animals were killed by CO_2_, and either the embryos were dissected from pregnant females or whole eyeballs were removed from postnatal animals. Tissues were then fixed in 10% neutral buffered paraformaldehyde overnight at 4°C, processed and embedded in paraffin. Serial sections were cut in 5-μm thickness through the midsection of the lens and stained with hematoxylin and eosin or used for subsequent experiments. Immunohistochemistry was performed as described previously [65] with the 3,3'-diaminobenzidine (DAB) kit (Vector Laboratories, Burlingame, CA, USA). Indirect immunohistological staining was conducted following standard procedures. Briefly, sections were washed twice in Tris-buffered saline (TBS) and blocked for 30 min with Image-iT™ FX signal enhancer (Molecular Probes). Then sections were washed twice in TBS before undergoing primary antibody incubation in 1% Bovine Albumin Fraction Solution (Invitrogen, Grand Island, NY, USA) and 0.1% (vol/vol) Triton X in TBS overnight at 4°C in a humidified chamber. After being washed twice in PBS, sections were incubated with the secondary antibody for 1 h at room temperature. Sections were then washed three times with PBS and mounted with fluorescence preserve mounting medium (Vector Laboratories). Primary antibodies were diluted (vol/vol) as follows: Brg1 (1:200), αA-crystallin (1:1,000), γ-crystallin (1:1,000), Flag (1:150), MIP26 (1:200) and Pax6 (1:400). Secondary antibodies were diluted as follows: Alexa 568 goat antirabbit (1:500) and biotin-conjugated secondary antirabbit (1:400). For immunofluorescence staining, primary antibodies were diluted in blocking solution and nuclei were stained with 4',6-diamidino-2-phenylindole (DAPI). Images were obtained with a Zeiss Axioskop II light microscope or a Leica MZ FLIII fluorescence stereomicroscope (Heerbrugg, Switzerland).

### Scanning and transmission electron microscopy

Three-month-old mouse lenses from wild-type and dnBrg1 animals were fixed overnight at room temperature in 0.08 M sodium cacodylate, 1.25% glutaraldehyde and 1% paraformaldehyde (pH 7.4). After fixation, the lens capsule and several of the outermost layers of the fiber cells were peeled off to show the fiber pattern. Then the lens samples were dehydrated through a graded series of ethanol and processed by critical point dry (CPD) using liquid carbon dioxide in a Tousimis Samdri 795 Critical Point Drier (Rockville, MD, USA). Subsequently, lens samples were transferred to a filter paper, placed in vacuum desiccators, mounted on a stub and sputter-coated for 2 min with gold-palladium in Denton Vacuum Desk-2 Sputter Coater (Cherry Hill, NJ, USA). Specimens were examined using a JEOL JSM6400 Scanning Electron Microscope (Peabody, MA, USA) with an accelerating voltage of 10 kV.

For transmission electron microscopy, eyes from 3-month-old mice were fixed for several hours in a 2% paraformaldehyde/2.5% glutaraldehyde solution. While in fixative, the posterior hemispheres of eyeballs were pierced with a fine needle. After being rinsed in 0.1 M cacodylate buffer, eyeballs were postfixed in a mixture of 1% OsO_4 _and 0.8% potassium ferrocyanide in 0.1 M cacodylate buffer for 2 h at 4°C. Specimens were then dehydrated in a graded series of ethanol and embedded in Epon (Serva, Heidelberg, Germany). Semithin sections (1 μm) were collected on uncoated glass slides and stained with methylene blue/azure II [66] for light microscopy. Ultrathin sections were mounted on uncoated copper grids, stained with uranyl acetate and lead citrate and examined on a Zeiss Libra electron microscope.

### Oligonucleotide microarrays and mRNA expression profiling

Lenses were isolated from day E15.5 transgenic and wild-type embryos and stored in RNA Later (Ambion, Woodlands, TX, USA). Newborn wild-type and *Hsf4*^*-/- *^lenses were described previously [49]. Whole eyeballs were isolated from newborn wild-type and conditional Brg1 knockouts as microdissected lenses were difficult to obtain because of their mechanical fragility. RNA isolations were performed using the RNeasy MiniElute Kit and RNase-Free DNase set (Qiagen, Valencia, CA, USA). RNA quality was assessed using the Agilent 2100 Bioanalyzer with the Nano LabChip Kit (Agilent Technologies, Palo Alto, CA, USA) following the manufacturer's instructions. Replicate sets of RNA from distinct day E15.5 dnBrg1 embryonic lenses (*n *= 4) and individual newborn eyeballs (*n *= 3) and corresponding numbers of stage-matched wild-type littermates were prepared for microarray analyses. cDNA synthesis and amplifications were performed with the Ovation™ RNA Amplification System V2 (Nugen, San Carlos, CA, USA) using 50 ng of total RNA per sample. Amplified cDNA were cleaned and purified with the DNA clean and Concentrator™-25 kit (Zymo Research, Orange, CA, USA). Fragmentation and labeling were performed using the FL Ovation™ cDNA Biotin Module V2 (Nugen) according to the manufacturer's instructions. The samples were subsequently hybridized on Mouse Genome 430A 2.0 arrays (Affymetrix, Santa Clara, CA, USA) following the manufacturer's specification.

### Bioinformatic tools and statistical filtering of RNA microarray results

Differentially regulated genes and/or mRNA between *dnBrg1 *and wild-type lenses were identified using biological quadruplicate sets (*n *= 4) of robust multichip average (RMA)-normalized Affymetrix CEL files [67] by a conjunction of Student's *t*-test (*P *< 0.05) and significance analysis of microarrays (SAM; false discovery rate FDR set to < 1%), using the TIGR Multiexperiment Viewer of the TM4 suite (Dana-Farber Cancer Institute, Boston, MA, USA) [68]. A similar strategy was used to identify differentially regulated genes and/or mRNA between *Hsf4*-null and wild-type lens (biological triplicates, RMA normalization, conjunction of *t*-test; *P *< 0.05 and SAM FDR < 5%). Primary data from this study were deposited in the National Center for Biotechnology Information (NCBI) Gene Expression Omnibus database under accession numbers GSE22322 (the dnBrg1 part), GSE22362 (the Hsf4 part) and GSE25168 (the Brg1 cKO part). The R-based extension to GeneSpring GS7 (Agilent Technologies, Santa Clara, CA, USA) was used to create a boxplot representation of 6,828 Brg1 target genes in Figure 8 to generate a five-number summary including the smallest observation, lower quartile, median, upper quartile, largest observation and indications of outlier observations. The GO and KEGG pathway functional annotations were performed using the National Institutes of Health web-based tool DAVID (Database for Annotation, Visualization and Integrated Discovery) [69]. GSEA (http://www.broad.mit.edu/gsea/) was additionally used to identify significantly enriched pathways disrupted in dnBrg1-transgenic lenses [46].

### qRT-PCR

Relative expression levels of 16 genes were verified by qRT-PCR. Total RNA from biological triplicates of transgenic and wild-type littermate lenses were isolated using RNeasy MiniElute Kit and RNase-Free DNase set (Qiagen, Valencia, CA, USA). Subsequently, cDNA was synthesized with Random Haxamer primers (Invitrogen) and Superscript TM III Reverse Transcriptase (Invitrogen) following the manufacturers' instruction. cDNA was diluted 10 times, and qRT-PCR was performed using the Applied Biosystems (ABI, Foster City, CA, USA) 7900HT fast Real-Time PCR system with Power SYBR Green PCR master mix (ABI). Specific primers for qRT-PCR are listed in Additional file 12. Primers were designed across neighboring introns using NCBI Primer-BLAST (http://www.ncbi.nlm.nih.gov/tools/primer-blast/index.cgi). Transcripts encoding β_2 _microglobulin (*B2M*), succinate dehydrogenase complex subunit A (*SDHA*), and hypoxanthine-guanine phosphoribosyltransferase (*HPRT*) genes were used for normalization of expression levels of both transgenic and wild-type results as described previously [45,70].

## A list of abbreviations used

**ATP**: adenosine-5'-triphosphate; **BAF**: Brg1 Associated Factor; **Brg1**: Brahma-Related Gene 1; **CREB**: cAMP Response Element Binding Factor; **DAVID**: Database for Annotation, Visualization and Integrated Discovery; **dn**: Dominant-negative; **GO**: Gene Ontology; **GSEA**: Gene Set Enrichment Analysis; **HMGA**1: High Mobility Group A 1; **Hsf4**: Heat Shock Transcription Factor 4; **INL**: Inner Nuclear Layer; **ISWI**: Imitation Switch; **KEGG**: Kyoto Encyclopedia of Genes and Genomes; **MIP**: Main Intrinsic Polypeptide; **Mitf**: Microphthalmia-associated Transcription Factor; **NES**: Normalized Enrichment Score; **NuRD**: Nucleosome Remodeling and Deacetylase; **OFZ**: Organelle Free Zone; **Pax6**: Paired Box Gene 6; **RAR**: Retinoic Acid Receptor; **RXR**: Retinoid X Receptor; SEM: scanning electron microscopy; **SWI/SNF**: Switch/Sucrose Nonfermentable; **Tbx2**:T-box Transcription Factor 2; TEM: transmission electron microscopy.

## Competing interests

The authors declare that they have no competing interests.

## Authors' contributions

SH carried out most of the experimental work, contributed to the bioinformatic analysis and drafted the manuscript. MKP performed all experiments to determine the expression pattern of Brg1 in the eye and significantly contributed to experimental work using the dominant-negative transgenic mice. WLW performed and analyzed the SEM experiments. LW and AN performed experiments with Hsf4-null lenses. BKC and KC developed the dominant-negative transgenic mouse model. ERT performed and analyzed the TEM studies. RAP, DM and PC participated in the Brg1 conditional knockout studies. JZ performed the bioinformatic and statistical analysis and participated in manuscript preparation. AC conceived the study and contributed to data analysis and manuscript preparation. All authors read and approved the final manuscript.

## Supplementary Material

Additional file 1 **Reduced expression of αA-crystallin and main intrinsic polypeptide (MIP), also known as aquaporin O and MIP26, in dominant-negative (dn) Brahma-related gene 1 (*Brg1*) (dnBrg1) transgenic lens**. Immunohistochemical staining using antibodies against **α**A-crystallin and MIP26 (aquaporin 0) revealed reduced expression of these two lens structural proteins in the dnBrg1 transgenic adult lenses. Note an evident lack of staining of both of the two proteins from the center of the lens, where the cataract is mainly initiated. Click here for file

Additional file 2 **Cell proliferation in the dnBrg1 lenses**. Immunohistochemical staining using antibodies against bromodeoxyuridine (BrdU) revealed no significant differences in lens epithelial cell proliferation in the embryonic day E15.5 lenses from wild-type **(A and C) **and dnBrg1 littermates **(B and D)**. Higher magnification of the anterior lenses of embryonic day E15.5 wild type **(C) **and dnBrg1 **(D) **are shown. Scale bar, 100 μm. Click here for file

Additional file 3 **Verification of microarray results by quantitative real-time polymerase chain reaction (qRT-PCR)**. Relative expression levels of Bfsp, Cdkn1b, Dnase2b, Dnmt3a, Fgfr1, Gsn, Hif1a, Hod, Jag1, Mab21l1, Pitpnm2, Prox1, Six3, Smarcd1, Smarce1 and Vim transcripts in wild-type (WT; shown in black) and dnBrg1 (shown in white) lenses were determined using qRT-PCR as described in Methods. β_2 _microglobulin (B2m), hypoxanthine-guanine phosphoribosyltransferase (Hprt) and succinate dehydrogenase complex subunit A (Sdha) transcripts were tested as internal references, and all were found unchanged between the wild-type and dnBrg1 lenses. The data are expressed relative to the unchanged expression level of B2m transcripts. Click here for file

Additional file 4 **A summary of the selected genes for qRT-PCR verification as potential candidate targets of Brg1**. Click here for file

Additional file 5 **Classification of significant genes into three categories: "Chromatin," "Lens Biology" and "Neuronal Function" following Gene Set Enrichment Analysis (GSEA)**. Upregulated (downregulated) genes are shown in red (blue), respectively. Curated gene sets, C2; GO gene sets, C5; Molecular Signature Database Class, MSigDB Class; normalized enrichment score, NES. Click here for file

Additional file 6 **Functional grouping of 178 genes that were commonly deregulated in both Pax6 heterozygous and dnBrg1 transgenic lenses using the Database for Annotation, Visualization and Integrated Discovery (DAVID)**. Upregulated genes, red; downregulated genes, blue. Click here for file

Additional file 7 **Loss of function of Brg1 via lens-specific deletion causes multiple ocular defects**. Compared to the control littermates **(A) **at postnatal day 21, lens-specific inactivation of Brg1 within the lens placode derivatives leaded to microphthalmia in the mutant mice **(B)**. The size of the P1 microdissected eyeballs **(D) **from the conditional knockout mutants was reduced, with a much smaller pupil opening (arrows) compared to the wild-type controls **(C)**. Click here for file

Additional file 8 **Analysis of the Le-Cre-driven deletion efficiency in Brg1 cKO**. **(A) **PCR analysis of genomic DNA prepared from newborn lens, cornea and tail. Detection of Brg1 deletion showed occasional germline deletion of Brg1 [52]: Lanes 1 and 2, lens; lane 3, tail; lane 4, cornea from two *Brg1*^*flox/flox*^; *Le-Cre *mice (M1 and M2) [7]. **(B) **PCR detection of *Brg1*^*flox*^, *Brg1 *WT and *cre *alleles. Lane 1-lens DNA from M1, which still showed *Brg1*^*flox *^band; two-tailed DNA from a *Brg1*^*flox/+ *^mouse; three-tailed DNA from M1 detected Cre expression. **(C) **Immunofluorescence localization of Brg1 in wild-type (*Brg1*^*flox/flox*^) and Brg1 cKO lenses (*Brg1*^*flox/flox*^; *Le-Cre*). Click here for file

Additional file 9 **Immunolocalization of αA-crystallin, γ-crystallin and Pax6 in Brg1 cKO**. Nuclei were shown by 4',6-diamidino-2-phenylindole (DAPI) staining (blue). Perturbed αA-crystallin (α-Cry) and **γ**-crystallin (γ-Cry) expression (red) was found in the Brg1 mutant lenses. Pax6 (red) is mainly expressed in the lens epithelial cells in both Brg1^flox/flox ^and Brg1 mutant P1 lenses. Scale bar, 100 μm. Click here for file

Additional file 10 **A list of 230 transcripts that were commonly deregulated in both dnBrg1 transgenic and Brg1 cKO lenses**. Upregulated genes, red; downregulated genes, blue. Click here for file

Additional file 11 **Identification of several GO categories "Biological Function" using the Database for Annotation, Visualization and Integrated Discovery (DAVID) that contain large numbers of genes disrupted in dnBrg1 transgenic lenses**. Upregulated genes, red; downregulated genes, blue. Click here for file

Additional file 12 **A list of primers used in qRT-PCR**. Click here for file
