# Supplementary figures and images for: Chromatin remodeling enzyme Brg1 is required for mouse lens fiber cell terminal differentiation and its denucleation
Source: Epigenetics Chromatin. 2010 Nov 30;3:21. doi: 10.1186/1756-8935-3-21 (PMC3003251; doi:10.1186/1756-8935-3-21)

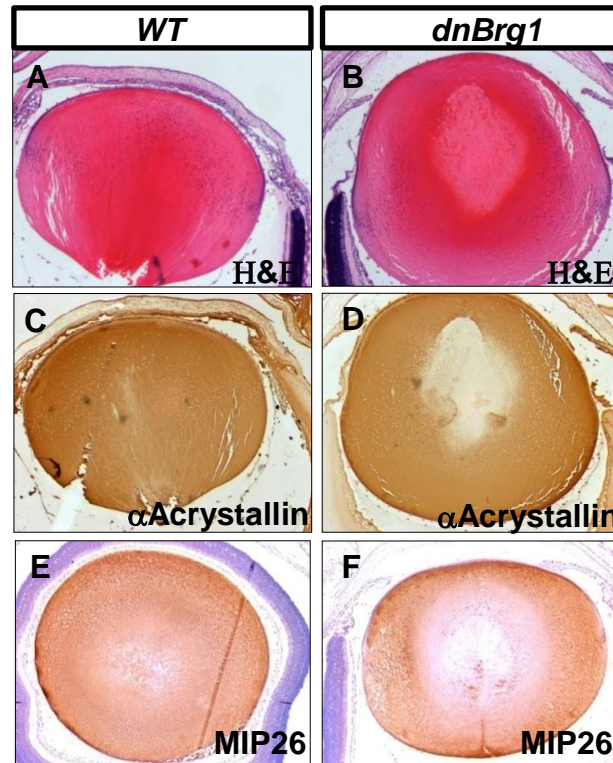

Supplement: Additional file 1 — Reduced expression of αA-crystallin and main intrinsic polypeptide (MIP), also known as aquaporin O and MIP26, in dominant-negative (dn) Brahma-related gene 1 (Brg1) (dnBrg1) transgenic lens. Immunohistochemical staining using antibodies against αA-crystallin and MIP26 (aquaporin 0) revealed reduced expression of these two lens structural proteins in the dnBrg1 transgenic adult lenses. Note an evident lack of staining of both of the two proteins from the center of the lens, where the cataract is mainly initiated. [file 1756-8935-3-21-S1.PDF]

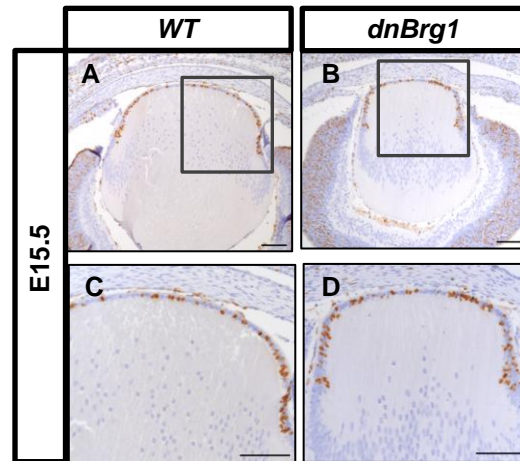

Supplement: Additional file 2 — Cell proliferation in the dnBrg1 lenses. Immunohistochemical staining using antibodies against bromodeoxyuridine (BrdU) revealed no significant differences in lens epithelial cell proliferation in the embryonic day E15.5 lenses from wild-type (A and C) and dnBrg1 littermates (B and D). Higher magnification of the anterior lenses of embryonic day E15.5 wild type (C) and dnBrg1 (D) are shown. Scale bar, 100 μm. [file 1756-8935-3-21-S2.PDF]

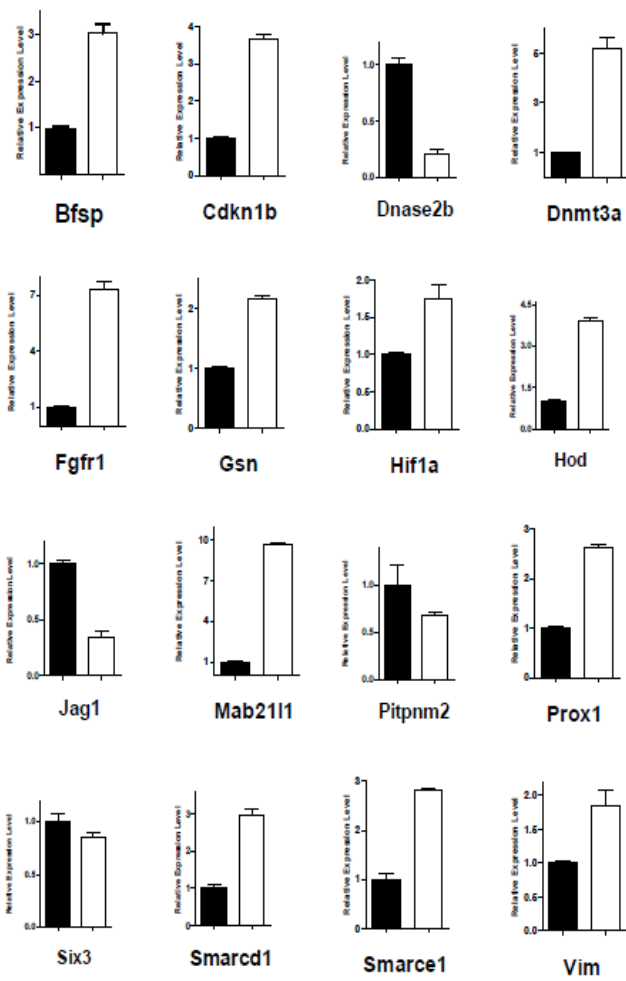

Supplement: Additional file 3 — Verification of microarray results by quantitative real-time polymerase chain reaction (qRT-PCR). Relative expression levels of Bfsp, Cdkn1b, Dnase2b, Dnmt3a, Fgfr1, Gsn, Hif1a, Hod, Jag1, Mab21l1, Pitpnm2, Prox1, Six3, Smarcd1, Smarce1 and Vim transcripts in wild-type (WT; shown in black) and dnBrg1 (shown in white) lenses were determined using qRT-PCR as described in Methods. β2 microglobulin (B2m), hypoxanthine-guanine phosphoribosyltransferase (Hprt) and succinate dehydrogenase complex subunit A (Sdha) transcripts were tested as internal references, and all were found unchanged between the wild-type and dnBrg1 lenses. The data are expressed relative to the unchanged expression level of B2m transcripts. [file 1756-8935-3-21-S3.PDF]

|     | <i>Brg1</i> flox/flox                                                                              | <i>Brg1</i> flox/flox; Le-Cre                                                                        |
|-----|----------------------------------------------------------------------------------------------------|------------------------------------------------------------------------------------------------------|
| P21 | <p><b>A</b></p> 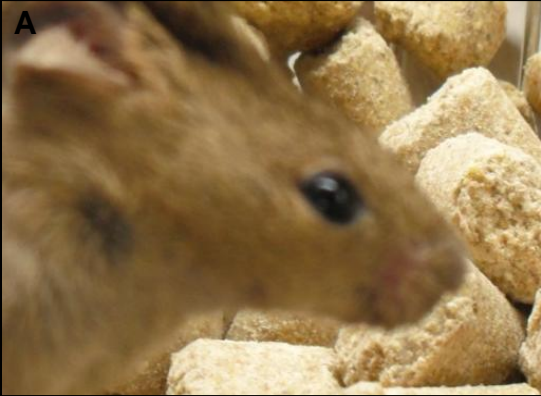  | <p><b>B</b></p> 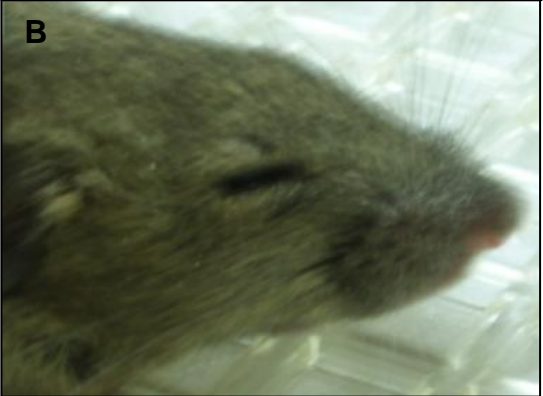  |
| P1  | <p><b>C</b></p> 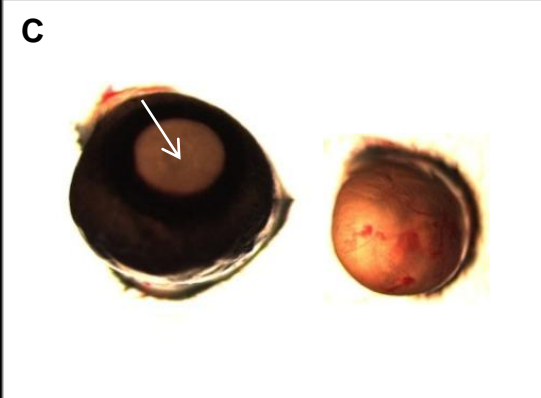 | <p><b>D</b></p> 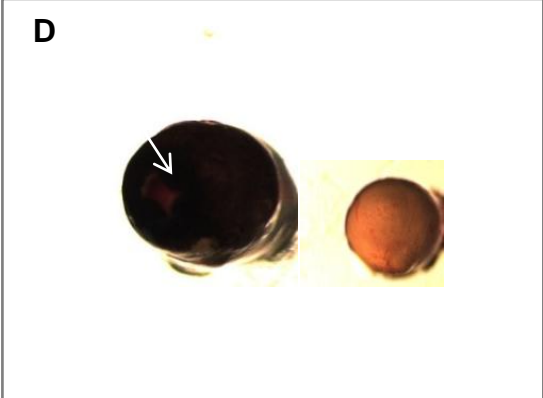 |

Supplement: Additional file 7 — Loss of function of Brg1 via lens-specific deletion causes multiple ocular defects. Compared to the control littermates (A) at postnatal day 21, lens-specific inactivation of Brg1 within the lens placode derivatives leaded to microphthalmia in the mutant mice (B). The size of the P1 microdissected eyeballs (D) from the conditional knockout mutants was reduced, with a much smaller pupil opening (arrows) compared to the wild-type controls (C). [file 1756-8935-3-21-S7.PDF]

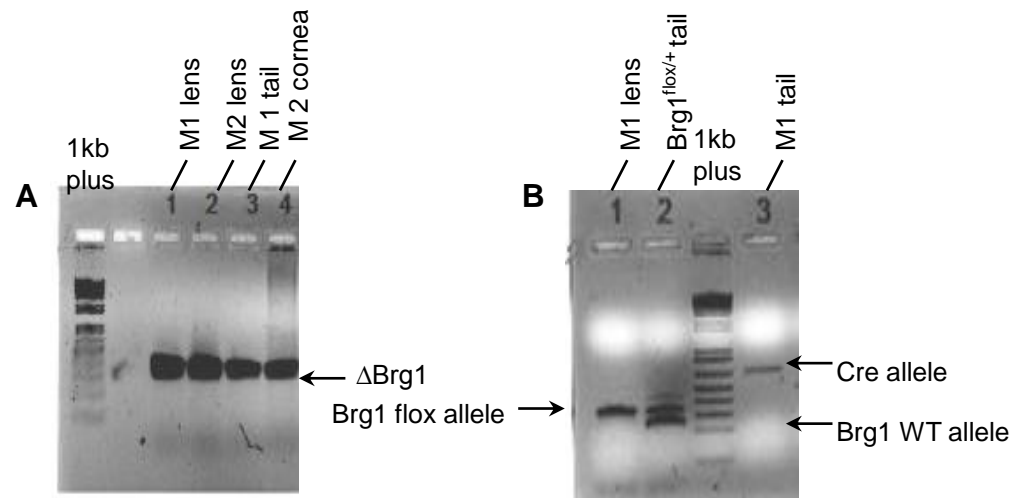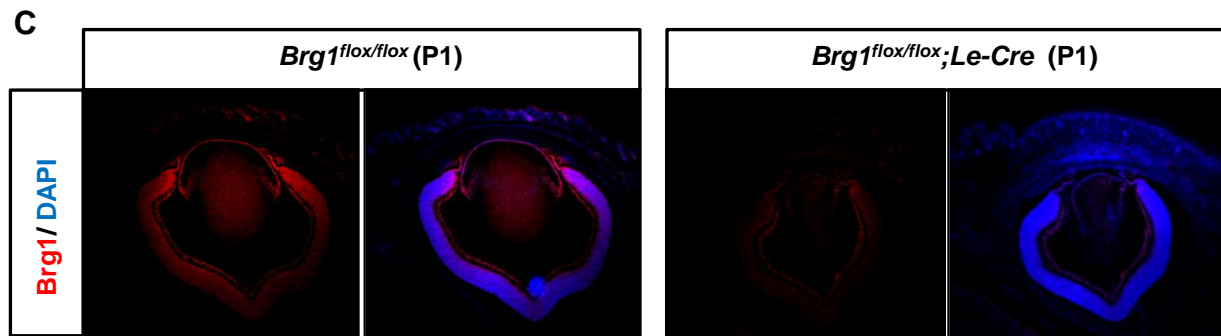

Supplement: Additional file 8 — Analysis of the Le-Cre-driven deletion efficiency in Brg1 cKO. (A) PCR analysis of genomic DNA prepared from newborn lens, cornea and tail. Detection of Brg1 deletion showed occasional germline deletion of Brg1 [52]: Lanes 1 and 2, lens; lane 3, tail; lane 4, cornea from two Brg1flox/flox; Le-Cre mice (M1 and M2) [7]. (B) PCR detection of Brg1flox, Brg1 WT and cre alleles. Lane 1-lens DNA from M1, which still showed Brg1flox band; two-tailed DNA from a Brg1flox/+ mouse; three-tailed DNA from M1 detected Cre expression. (C) Immunofluorescence localization of Brg1 in wild-type (Brg1flox/flox) and Brg1 cKO lenses (Brg1flox/flox; Le-Cre). [file 1756-8935-3-21-S8.PDF]

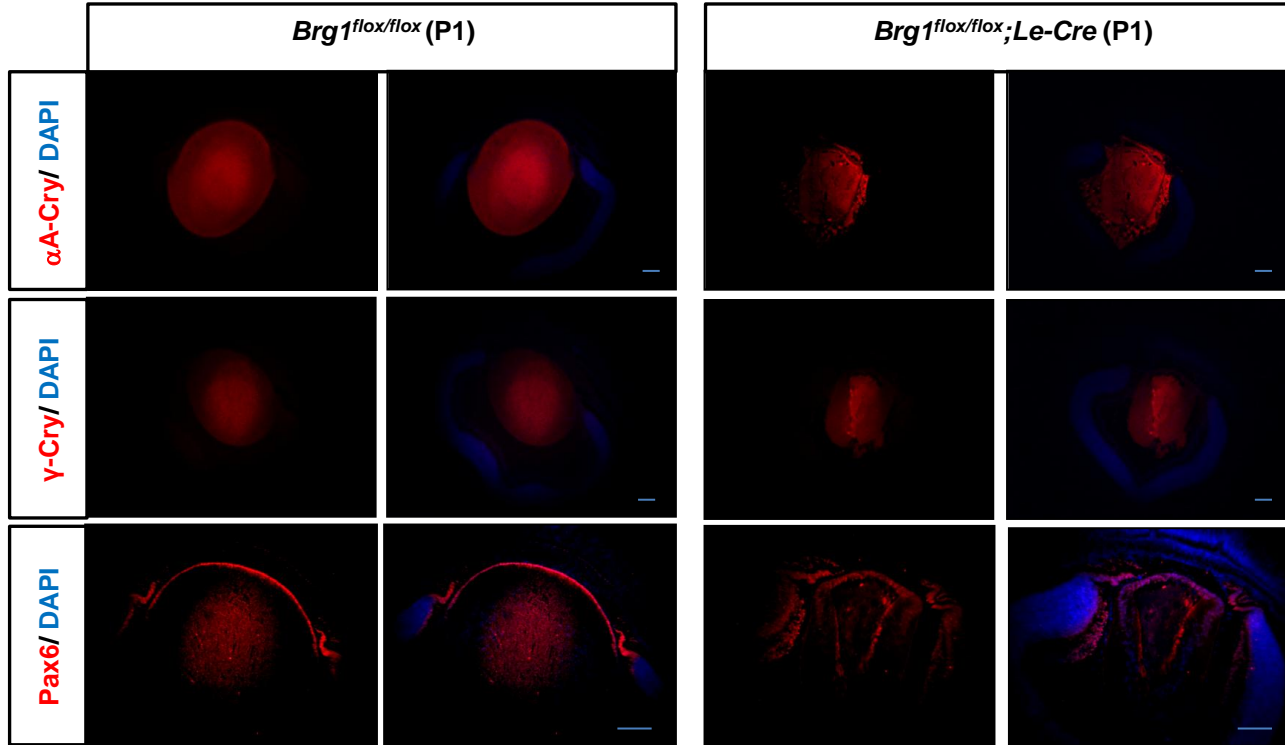

Supplement: Additional file 9 — Immunolocalization of αA-crystallin, γ-crystallin and Pax6 in Brg1 cKO. Nuclei were shown by 4',6-diamidino-2-phenylindole (DAPI) staining (blue). Perturbed αA-crystallin (α-Cry) and γ-crystallin (γ-Cry) expression (red) was found in the Brg1 mutant lenses. Pax6 (red) is mainly expressed in the lens epithelial cells in both Brg1flox/flox and Brg1 mutant P1 lenses. Scale bar, 100 μm. [file 1756-8935-3-21-S9.PDF]
