# Supplementary material for: Chromatin remodeling enzyme Brg1 is required for mouse lens fiber cell terminal differentiation and its denucleation
Source: Epigenetics Chromatin. 2010 Nov 30;3:21. doi: 10.1186/1756-8935-3-21 (PMC3003251; doi:10.1186/1756-8935-3-21)
Supplement: Additional file 4 — A summary of the selected genes for qRT-PCR verification as potential candidate targets of Brg1. [file 1756-8935-3-21-S4.PDF]

A summary of selected genes for qRT-PCR verification as potential candidate targets of Brg1

1450571_a_at	Bfsp1	beaded filament structural protein in lens-CP94
1419497_at	Cdkn1b	cyclin-dependent kinase inhibitor 1B (P27)
1450237_at	Dnase2b	deoxyribonuclease II beta
1423066_at	Dnmt3a	DNA methyltransferase 3A
1425911_a_at	Fgfr1	fibroblast growth factor receptor 1
1415812_at	Gsn	gelsolin
1448183_a_at	Hif1a	hypoxia inducible factor 1, alpha subunit
1451776_s_at	Hod	RIKEN cDNA 2300002F06 gene
1421105_at	Jag1	jagged 1
1421369_a_at	Mab21l1	mab-21-like 1 (C. elegans)
1419757_at	Pitpnm2	retinal degeneration B2 homolog (Drosophila)
1421336_at	Prox1	prospero-related homeobox 1
1426638_at	Six3	sine oculis-related homeobox 3 homolog (Drosophila)
1448913_at	Smarcd1	SWI/SNF related, matrix associated, actin dependent regulator of chromatin, subfamily d, member 1
1422675_at	Smarce1	SWI/SNF related, matrix associated, actin dependent regulator of chromatin, subfamily e, member 1
1450641_at	Vim	vimentin
