# Supplementary material for: Chromatin remodeling enzyme Brg1 is required for mouse lens fiber cell terminal differentiation and its denucleation
Source: Epigenetics Chromatin. 2010 Nov 30;3:21. doi: 10.1186/1756-8935-3-21 (PMC3003251; doi:10.1186/1756-8935-3-21)
Supplement: Additional file 5 — Classification of significant genes into three categories: "Chromatin," "Lens Biology" and "Neuronal Function" following Gene Set Enrichment Analysis (GSEA). Upregulated (downregulated) genes are shown in red (blue), respectively. Curated gene sets, C2; GO gene sets, C5; Molecular Signature Database Class, MSigDB Class; normalized enrichment score, NES. [file 1756-8935-3-21-S5.PDF]

Classification of significant genes into three categories: “Chromatin”, “Lens Biology” and “Neuronal Function” following Gene Set Enrichment Analysis (GSEA). Up-regulated (down-regulated) genes are shown in red (blue), respectively. Curated gene sets, C2; GO gene sets, C5; Molecular Signature Database Class, MSigDB Class; normalized enrichment score, NES.

Category	MSigDB Class	NAME	NES	SIGNIFICANT GENES (CORE ENRICHMENT)
	C5	TRANSCRIPTION_FACTOR_COMPLEX	1.54	EDF1, HDAC3, TAF9, ING2, TRRAP, TAF11, RUVBL1, TAF12, SAP18, GTF2H1, SUB1, FOXF2, HDAC2, NARG1, ACTL6A, HDAC5
	C2	HDACPATHWAY	0.96	CALM1, PPP3CC, CAMK1, HDAC5, MEF2A, NFATC2, IGF1R
	C5	DAMAGED_DNA_BINDING	-1.28	FEN1, XPC, MPG, POLH, ERCC4,
	C5	CHROMATIN	-1.29	TOP2B, FOXC1, JUNB, MAF, CBX1, H2AFY, INCENP, MCM2, KLHDC3, PAM, UPF1
Chromatin	C5	TRANSCRIPTION_FACTOR_ACTIVITY	-1.30	FOXL1, EGR2, ATF1, YEATS4, NANOG, ATF3, RFX3, MYBL2, FOSL2, NFATC1, ARNT2, TAF13, MNT, POU3F2, AR, GATA2, GATA3, PTTG1, XBP1, CCRN4L, PHF1, GATA1, BACH1, MBD1, FOXN2, TRIM28, NPAS2, MYEF2, AHR, HIC1, GLI2, PPARG, MYNN, RUNX1T1, RELA, NR2C2, POU3F1, EPAS1, LZTR1, AEBP1, NFIC, ETV5, SPI1, CREB1, SNAPC2, KLF9, NEUROG1, HDAC1, DLX3, STAT6, NFKB2, UBP1, GLI3, HSF1, SCMH1, DLX2, LMO1, SOX4, CREBBP, TBX1, HNF4A, TCFL5, E2F3, JARID1B, TSC22D3, SREBF1, GTF2H4, THRA, ZBTB48, FLI1, SCAND1, ZBTB17, HSF2
	C5	HISTONE_MODIFICATION	-1.31	MYST3, MAP3K12, NSD1, CREBBP, PPARGC1A
	C5	CHROMATIN_BINDING	-1.51	CENPA, CBX1, NSD1, CBX3, BCL6, SMC1A, KLHDC3, PAM, UPF1, CDCA5, POLD1
	C2	GLUTATHIONE_METABOLISM	1.53	MGST3, GGT1, GSTT2, GSTM1, IDH2, GCLM, GPX4, GSTZ1, GSTM5
	C5	ANTIOXIDANT_ACTIVITY	1.45	MGST3, GPX4, GSTZ1, CYGB
	C5	GLUTATHIONE_TRANSFERASE_ACTIVITY	1.38	MGST3, GSTT2, GSTM1, GSTZ1, GSTM5,
	C2	RHOPATHWAY	1.23	ARPC1B, ARPC3, ARPC1A, PFN1, PIP5K1B, ARHGAP5, PIP5K1A
	C2	P38MAPKPATHWAY	1.05	MAPKAPK2, MAPKAPK5, HSPB2, STAT1, DDIT3, DAXX, MEF2A, MYC, MAPK14, HRAS, MAX
	C2	HSA04540_GAP_JUNCTION	1.03	PDGFB, PDGFRB, RAF1, PLCB4, TUBB2C, MAP2K5, TUBB3, MAP2K2, ADCY9, ADCY6, TUBB2A, PLCB1, MAPK1, ADCY4, GNAI3, GUCY1B3, EDG2, PRKACA, HRAS, PDGFD, EGF, GUCY1A3
	C2	HSA04140_REGULATION_OF_AUTOPHAGY	0.98	PIK3C3, GABARAPL1
	C2	HSA04310_WNT_SIGNALING_PATHWAY	0.93	TP53, CTBP2, PPP3CC, CCND3, PLCB4, CACYBP, RUVBL1, LRP6, WNT16, PPP2R2B, PPP2CB, PORCN, FZD2, NFATC2, MYC, SMAD2, DKK2, FZD7, PLCB1, GSK3B, AXIN1, CUL1
	C2	GSK3PATHWAY	-1.03	WNT1, MYD88, GNAI1, CD14, PPP2CA, TIRAP, IRAK1, AKT1, PIK3CA, RELA, TLR4, PIK3R1
	C2	SIG_REGULATION_OF_THE_ACTIN_CYTOSKELETON_BY_RHO_GTPASES	-1.03	PAK1, FSCN1, WASL, GDI2, FSCN3, LIMK1, PAK3, RHO, ACTG2, CFL1, ACTR2, MYLK, AKT1, GDI1, WASF1
	C2	INTEGRINPATHWAY	-1.06	ACTN3, RAP1A, MAPK8, ACTN2, SRC, TLN1, BCAR1
Lens biology	C2	ST_P38_MAPK_PATHWAY	-1.11	HSPB1, MKNK2, ATF1, DUSP10, IL1R1, MKNK1, MYEF2, AKT1, MAPK12, NR2C2, CREB1, SRF, CREB3
	C2	HSA04340_HEDGEHOG_SIGNALING_PATHWAY	-1.11	BMP4, WNT8A, CSNK1D, BMP2, WNT9B, WNT10A, GAS1, WNT6, GLI1, PRKX, DHH, HHIP, ZIC2, CSNK1E, GLI2, WNT5A, BTRC, GLI3, WNT5B, SMO
	C2	HSA04150_MTOR_SIGNALING_PATHWAY	-1.21	HIF1A, ULK2, RPS6KA1, AKT3, PIK3CG, RPS6KA2, FIGF, RPS6, ULK1, AKT1, PIK3CA, RPS6KA3, TSC1, GBL, PIK3R2, PIK3R1, AKT2, VEGFB, EIF4EBP1
	C5	MAPKKK_CASCADE_GO_0000165	-1.24	CHRNA7, MAP2K4, C5AR1, MAPK10, GADD45B, MDFIC, FPR1, MAP4K5, GHRL, MAP3K11, PTPLAD1, DUSP2, SPRED1, GPS1, ADRB2, GPS2, MBIP, RGS4, DUSP10, NF1, SPRED2, MAP3K12, SCG2, CD81, CARTPT, MAPK8, DUSP8, TPD52L1, MAP2K7, FGF13, PLCE1, CXCR4, MAP3K3, MAPK8IP2, DUSP6, ATP6AP2, CCM2, NRTN, MINK1, SH2D3C, CAMKK2
	C2	ST_INTEGRIN_SIGNALING_PATHWAY	-1.25	MAP3K11, AKT3, PAK3, SOS2, RHO, ACTN1, TERF2IP, PKLR, ARHGEF7, ITGB3BP, ACTR2, MAPK8, WAS, MYLK, AKT1, MAP2K7, EPHB2, PIK3CA, RALA, MAPK8IP2, ITGA5, ITGA4, AKT2, PLCG2, SRC, TLN1, ITGA3, ABL1, BCAR1
	C5	REGULATION_OF_CELL_DIFFERENTIATION	-1.25	ETS1, SCIN, SOCS5, TBX5, INHBA, SART1, NANOG, NF1, CARTPT, CNTN4, NOTCH4, SNF1LK, BOC, PPARG, NOTCH1, CDK6, SPI1, TBX3, NOTCH2, ZBTB16, DTX1, TCFL5
	C5	RHO_PROTEIN_SIGNAL_TRANSDUCTION	-1.31	FGD1, FGD2, LIMK1, CENTD3, ARHGDIB, RHOA, ALS2, ABCA1, CFL1, FGD4, TSC1, ARHGDIA, ARHGAP4, RHOG, RTKN, APOA1, ARHGDIG
	C2	ST_ERK1_ERK2_MAPK_PATHWAY	-1.40	RPS6KA1, RPS6KA2, SOS2, MKNK2, ATF1, MKNK1, RAP1A, TRAF3, RPS6KA3, CREB1, DUSP6, BAD, CREB3
	C2	HSA04330_NOTCH_SIGNALING_PATHWAY	-1.55	HES1, JAG2, DVL2, MAML1, NOTCH4, NOTCH1, DLL4, PSENEN, NCOR2, HDAC1, MFNG, NOTCH2, DTX3, DLL3, CREBBP, DTX1, DTX2, JAG1
Neuronal function	C5	NERVOUS_SYSTEM_DEVELOPMENT	-1.24	ACCN1, ACHE, ALS2, APLP1, ARNT2, ARTN, ATN1, ATP2B2, AVIL, BAI1, BDNF, BPNT1, BTG4, CBLN1, CDK5R1, CDK6, CHERP, CLN8, CNTN4, CNTN6, COL4A4, CRMP1, CTF1, CYFIP1, CYP46A1, DCX, DLX2, DPYSL3, DPYSL4, DPYSL5, DSCAM, DTX1, DVL3, EGR2, EIF2B4, EPHB2, FABP7, FGF13, FGF14, FGF5, GFRA3, GJB1, GLI2, GLRB, GPR56, GREM1, HES1, KALRN, KCNQ2, L1CAM, LHX1, LMX1B, LST1, LY6H, MAP1S, MAPT, MBP, MSI1, MYLIP, NAB2, NCOA6, NEUROG1, NF1, NF2, NHLH1, NINJ1, NMUR2, NPAS2, NPTX1, NR2C2, NRTN, NTNG1, OLFM1, OPHN1, PARK2, PBX1, PCDH1, PCDH18, PCDHA10, PCDHB12, PCDHB14, PCDHB17, PCDHB3, PCDHB9, PDGFC, PICK1, PITPNM1, POU3F3, PSPN, PTPRZ1, ROBO1, RPS6KA3, SCN8A, SERPINI1, SH3GL2, SH3GL3, SIM2, SLIT1, SMPD1, SNCA, SOX11, SOX3, SPOCK1, ST8SIA2, STMN3, THY1, TPP1, TRIM3, UNC5C, ZBTB16, ZIC2,
	C2	HSA01510_NEURODEGENERATIVE_DISEASES	-1.27	ALS2, NEFH, GFAP, MAPT, SNCA, BCL2L1, PARK7, ATN1, CASP6, NGFR, BAD, CREBBP, APLP1, PARK2
