# Supplementary material for: Chromatin remodeling enzyme Brg1 is required for mouse lens fiber cell terminal differentiation and its denucleation
Source: Epigenetics Chromatin. 2010 Nov 30;3:21. doi: 10.1186/1756-8935-3-21 (PMC3003251; doi:10.1186/1756-8935-3-21)
Supplement: Additional file 6 — Functional grouping of 178 genes that were commonly deregulated in both Pax6 heterozygous and dnBrg1 transgenic lenses using the Database for Annotation, Visualization and Integrated Discovery (DAVID). Upregulated genes, red; downregulated genes, blue. [file 1756-8935-3-21-S6.PDF]

Functional grouping of 178 genes that were commonly deregulated in both Pax6 heterozygous and dnBrg1 transgenic lenses using the Database for Annotation, Visualization and Integrated Discovery (DAVID)

Category	Term	Count	%	p value	Genes
GO TERM BIOLOGICAL PROCESS	GO:0006508~proteolysis	7	8.33	0.332	TPP1, ATG4B, NEDD4L, USP48, RNF34, USP43, PCSK1N
	GO:0006259~DNA metabolic process	6	7.14	0.043	PRIM1, RAD51C, POLH, RRM1, RBMS1, DNASE2B
	GO:0055085~transmembrane transport	5	5.95	0.158	SLC2A8, AI132487, AQP1, HCN3, ABCC5
	GO:0030163~protein catabolic process	5	5.95	0.248	ATG4B, NEDD4L, USP48, RNF34, USP43
	GO:0045184~establishment of protein localization	5	5.95	0.351	FAM160A2, ATG4B, HGS, KPNA3, MYO5B
	GO:0006260~DNA replication	4	4.76	0.032	PRIM1, POLH, RRM1, RBMS1
	GO:0019725~cellular homeostasis	4	4.76	0.206	MAFG, ANXA7, GAA, RYR2
	GO:0006511~ubiquitin-dependent protein catabolic process	2	2.38	0.477	USP48, USP43
	GO:0032774~RNA biosynthetic process	2	2.38	0.439	PRIM1, SIRT7
	GO:0051052~regulation of DNA metabolic process	2	2.38	0.233	GMNN, CD40
GO TERM MOLECULAR FUNCTION	GO:0000166~nucleotide binding	20	23.81	0.005	RAD51C, CARS, SPHK2, CTPS2, SIRT7, ELAVL4, DAPK2, ABCG4, DDX4, RND2, RRM1, PTBP2, HCN3, MYO5B, ABCC5, MYO5C, RDM1, DNM2, RBMS1, TUBB4
	GO:0008270~zinc ion binding	13	15.48	0.290	CARS, ZFP385A, LMX1B, SIRT7, TTC3, PRIM1, ZDHHC14, SALL4, ZFP790, HGS, RNF34, RNF121, ZFP276
	GO:0003677~DNA binding	10	11.90	0.474	MAFG, RAD51C, SALL4, LMX1B, POLH, NFATC2, ZFP276, TFB1M, RBMS1, RDM1
	GO:0003723~RNA binding	6	7.14	0.213	CARS, PNPT1, PTBP2, ELAVL4, RBMS1, RDM1
	GO:0004518~nuclease activity	3	3.57	0.166	RAD51C, PNPT1, DNASE2B
	GO:0004520~endodeoxyribonuclease activity	2	2.38	0.060	RAD51C, DNASE2B
GO TERM CELLULAR COMPARTMENT	GO:0044421~extracellular region part	6	7.14	0.326	LPL, PRRT1, SPOCK1, PCSK1N, SPON1, CCL6
	GO:0044430~cytoskeletal part	6	7.14	0.326	ATG4B, MYO5B, DNM2, TUBB4, RANBP10, MYO5C
	GO:0005773~vacuole	4	4.76	0.077	TPP1, GAA, TTC3, DNASE2B
	GO:0015630~microtubule cytoskeleton	4	4.76	0.377	ATG4B, DNM2, TUBB4, RANBP10
KEGG PATHWAY	mmu00240:Pyrimidine metabolism	4	4.76	0.009	PRIM1, PNPT1, RRM1, CTPS2
	mmu04142:Lysosome	3	3.57	0.100	TPP1, GAA, DNASE2B
	mmu00480:Glutathione metabolism	3	3.57	0.023	GSTA3, GGT7, RRM1
	mmu04020:Calcium signaling pathway	2	2.38	0.586	SPHK2, RYR2
	mmu04370:VEGF signaling pathway	2	2.38	0.294	SPHK2, NFATC2
