# Supplementary material for: Chromatin remodeling enzyme Brg1 is required for mouse lens fiber cell terminal differentiation and its denucleation
Source: Epigenetics Chromatin. 2010 Nov 30;3:21. doi: 10.1186/1756-8935-3-21 (PMC3003251; doi:10.1186/1756-8935-3-21)
Supplement: Additional file 11 — Identification of several GO categories "Biological Function" using the Database for Annotation, Visualization and Integrated Discovery (DAVID) that contain large numbers of genes disrupted in dnBrg1 transgenic lenses. Upregulated genes, red; downregulated genes, blue. [file 1756-8935-3-21-S11.PDF]

Identification of several GO categories "Biological Function" using the Database for Annotation, Visualization and Integrated Discovery (DAVID) that contain large numbers of genes disrupted in dnBrg1 transgenic lenses.

Category	Term	Count	%	Genes (RED = upregulated; BLUE = downregulated)
GOTERM_BP	GO:0006281~DNA repair	47	2.28%	Xpa, Trpc2, H2afx, Rad51ap1, Blm, Kin, Apex1, Parp1, Giyd2, Bccip, Sfpq, Trp53, Hmgb2, Trex1, Slk, Atm, Xrcc4, Exo1, Ube2a, Rad21, Rad51c, Polb, Xab2, C230052I12Rik, Rad51l1, Ercc8, Vcp, Shfm1, Atrx, Gtf2h1, Ankrd17, Smc5, Nsmce1, Gtf2h4, Rad18, Ercc4, Fancc, Supt16h, Rad23a, Ddb2, Usp1, Xrn2, Ssrp1, Smc1a, Smc3, Esco1, Prpf19,
		27	1.34%	Pold1, Pms2, Mlh3, Ogg1, Polh, Mpg, Xpc, Slk, Msh2, Sod2, Fen1, Ercc8, Polg2, Atrx, Nsmce1, Gtf2h4, Fancg, Ercc4, Tnfaip1, Rad23b, Fanca, Upf1, Ssrp1, Smc1a, Smc6, Mus81, Fancm,
GOTERM_BP	GO:0006325~establishment and/or maintenance of chromatin architecture	43	2.09%	Ing2, Hltf, H1f0, H2afx, Smarcd1, Baz1b, Hist3h2ba, Hdac2, Smarca4, Rbl2, Prmt5, Foxp3, Smarcb1, Brd8, Mbd3, Rnf20, Smarca2, Eed, Hmgb2, Hmg20a, Ruvbl1, Smarcd3, Ttf1, Hist1h1c, Rnf2, Dnmt3a, Sap18, Smarcc1, Actl6a, Trrap, Ncor1, Gpx4, Noc2l, Hdac3, Men1, Tmem38b, Smarca5, Smarce1, Arid4b, Hira, Banp, Rcor1, Chd4,
		26	1.29%	Chd1, Actl6b, Nsd1, Tlk2, Sirt7, Hnf1a, Hist1h1c, Hist1h3i, Sap18, Mcm2, Dapk3, Suv420h1, Hdac11, H1fx, Tbl1xr1, Chd6, H2afj, Hira, Bptf, Hmg20b, Sox3, Asf1b, Huwe1, Cbx1, Mphosph8, Crebbp,
GOTERM_BP	GO:0006512~ubiquitin cycle	84	4.08%	Rnf138, Map1lc3a, Cdc23, Usp36, Amfr, Rnf41, Rnf25, Senp1, Rfwd2, Fbxo9, Cacybp, Rnf20, Usp15, Uchl5, Tbl1x, Pja1, Ufc1, Cdc26, Anapc4, Rwdd3, Rnf128, Ube2s, Fbxw2, Sae1, Fbxl6, Spop, Rnf2, Skp2, Usp2, Arih1, Ube2l3, Wdsub1, Rnf19b, Gtpbp4, Rwdd1, Fbxo4, Topors, Anapc5, Usp1, Atg5, Zc3hc1, Usp20, Prpf19, Ube2z, Usp48, Itch, Fbxo22, Otub1, Socs3, Trip12, Tomm22, Gclc, Senp6, Pias4, Lrrc41, Asb8, Fbxl12, Rnf7, Usp8, Usp38, Rnf6, Cdc20, Ube2a, Cyhr1, Fbxw5, Rnf14, Cul3, Vcp, Ube2g2, Rnf167, Ubqln1, Cand1, Ube2i, Rad18, Ube2f, Anapc1, Rad23a, Birc6, Ppil2, Brcc3, Cdc34, Stub1, Sugt1, Bre,
		69	3.42%	Hectd3, Fbxo42, Usp9x, Ube3a, Otub2, Usp19, Eif2ak4, Rnf11, Pja1, Ube2k, Znrf1, Rnf181, Rwdd2b, Znrf2, Usp2, Asb6, Ubac1, Vps11, Cbll1, Usp3, Mib2, Atg4b, Cuedc2, Dda1, Tbl1xr1, Topors, Anapc5, Ubl7, Ufm1, Siah2, Otud7a, Huwe1, Syt4, Nosip, Kcmf1, Usp12, Usp48, Otub1, Socs3, Usp22, Map1lc3b, Klhl13, Park2, Tulp4, Nedd4l, Fbxw4, Usp50, Uevld, Atg10, Rab40c, Aktip, Spsb4, Cyhr1, Ube2j1, Ube4a, Cand1, Socs4, Ube2r2, Usp29, Asb13, Ube2f, Rad23b, Anapc2, Birc6, Rabgef1, March2, Brcc3, Btrc, Fbxo3,
GOTERM_BP	GO:0006974~response to DNA damage stimulus	59	2.86%	Gadd45a, Timeless, Rad51ap1, Kin, Apex1, Giyd2, Parp1, Apc, Bccip, Sfpq, Trex1, Frag1, Rad21, Polb, Xab2, C230052I12Rik, Ercc8, Shfm1, Gtf2h1, Gtf2h4, Topors, Ercc4, Fancc, Supt16h, Usp1, Smc3, Esco1, Prpf19, Xpa, Trpc2, H2afx, Triap1, Btg2, Blm, Hipk2, Trp53, Hmgb2, Npm1, Pcbp4, Slk, Atm, Exo1, Xrcc4, Ube2a, Rad51c, Rad51l1, Vcp, Atrx, Ankrd17, Smc5, Nsmce1, Rad18, Rad23a, Ddb2, Xrn2, Ssrp1, Brcc3, Smc1a, Bre,
		32	1.58%	Pold1, Pms2, Mlh3, Chek2, Ogg1, Tlk2, Hipk2, Polh, Mpg, Xpc, Slk, Msh2, Sod2, Fen1, Ercc8, Polg2, Atrx, Nsmce1, Gtf2h4, Topors, Fancg, Ercc4, Tnfaip1, Rad23b, Upf1, Fanca, Ssrp1, Smc1a, Brcc3, Smc6, Mus81, Fancm,
GOTERM_BP	GO:0016568~chromatin modification	34	1.65%	Ing2, Hltf, Smarcd1, Baz1b, Hdac2, Smarca4, Rbl2, Prmt5, Smarcb1, Brd8, Foxp3, Mbd3, Rnf20, Eed, Hmg20a, Ruvbl1, Smarcd3, Ttf1, Rnf2, Dnmt3a, Smarcc1, Sap18, Actl6a, Trrap, Ncor1, Noc2l, Hdac3, Men1, Smarca5, Smarce1, Hira, Banp, Rcor1, Chd4,
		15	0.74%	Sap18, Dapk3, Nsd1, Suv420h1, Hdac11, Tlk2, Tbl1xr1, Hira, Sirt7, Bptf, Hmg20b, Hnf1a, Asf1b, Huwe1, Crebbp,
KEGG_PATHWAY	mmu03050:Proteasome	18	0.87%	Psmb2, Psmd11, Psmd7, Psmb7, Psmd8, Psmc3, Psmd14, Psmb3, Psma1, Psmb6, Psmd13, Psma4, Psmb1, Psma7, Psmd4, Psma5, Psmc1, Psma2,
KEGG_PATHWAY	mmu04120:Ubiquitin mediated proteolysis	32	1.55%	Ube2z, Cdc23, Itch, Trim37, Rfwd2, Socs3, Trip12, Cdc26, Pias4, Anapc4, Rnf7, Ube2s, Sae1, Skp2, Cdc20, Ube2a, Ube2l3, Ercc8, Cul3, Ube2g2, Fbxo4, Ube2i, Keap1, Anapc5, Ube2f, Anapc1, Ddb2, Birc6, Ppil2, Cdc34, Stub1, Prpf19,
		16	0.79%	Ercc8, Ube2j1, Socs3, Ube3a, Ube4a, Ube2r2, Ube2k, Klhl13, Anapc5, Ube2f, Park2, Anapc2, Nedd4l, Birc6, Huwe1, Btrc,
