# Supplementary material for: Chromatin remodeling enzyme Brg1 is required for mouse lens fiber cell terminal differentiation and its denucleation
Source: Epigenetics Chromatin. 2010 Nov 30;3:21. doi: 10.1186/1756-8935-3-21 (PMC3003251; doi:10.1186/1756-8935-3-21)
Supplement: Additional file 12 — A list of primers used in qRT-PCR. [file 1756-8935-3-21-S12.PDF]

Gene	Primers
B2m	5´ -TGGTGCTTGTCTCACTGACC-3´<br>5´ -TATGTTCGGCTTCCCATTCT-3´
Bfsp	5´ -GTTACTGGGATGAGGGAGGAG-3´<br>5´ -CTGCAGCTCAGCTTTCTGTG -3´
Cdkn1b	5´ -TCAAACGTGAGAGTGTCTAACG -3´<br>5´ -CCGGGCCGAAGAGATTTCTG -3´
Dnase2b	5´-GCCCAGGGTCTAAACTTCGT-3´<br>5´-TTCTGCCAGGTTTGTGCTAA-3´
Dnmt3a	5´ -GTGCAGAAACATCGAGGACA -3´<br>5´ -ATGCCTCCAATGAAGAGTGG -3´
Fgfr1	5’-GTTTAAGCCTGACCACCGAA-3’<br>5’-GAAGGCACCACAGAATCCAT-3’
Gsn	5’-TCCGGCTACTTCAAGTCTGG-3’<br>5’-CTCTGGACCACCACCTCATT-3’
Hprt	5´ -GTTGTTGGATATGCCCTTGA-3´<br>5´ -GGCTTTGTATTTGGCTTTTCC-3´
Hif1a	5´ -TTCTCAGTCGACACAGCCTC-3´<br>5´ - CCAAAAGTTCTTCCGGCTC-3´
Hod	5´ -CAACAAGGTCAACAAGCACC -3´<br>5´ -AACCATTTCTGCGTCTGCTC -3´
Jag1	5´ - GTCCCAAGCATGGGTCTTGT-3´<br>5´ - TGCACTTGTCGCAGTACAGG-3´
Mab21l1	5´ - CAGGAACCGCGTTTCATCAG-3´<br>5´ - CAGGAACCGCGTTTCATCAG-3´
Pitpnm2	5´ -GCTAGTCCTAGCCTTGAGGAAA-3´<br>5´ -TTGTACACTTGCTGACACGCT-3´
Prox1	5´ -TCCGACATCTCACCTTATTCAG-3´<br>5´ -AACATGAGTTTTGCCTTTTTCAA-3´
Sdha	5 ´-GAGGAAGCACACCCTCTCATA-3 ´<br>5 ´-GCACAGTCAGCCTCATTCAA-3 ´
Six3	5’ -CCCGGCTTCTCTTACCTTTCT- 3’<br>5’ –GAATCGGCGAAGTTTGGCAAC- 3’
Smarca2	5´ -CCACCAAGTCTGAAGATCGTG- 3’<br>5´ -CCGCCTGAAGATTTAAGCCCA- 3’
Smarca4	5´ -TGCTGAAGGACAGACACCTG-3´<br>5´ -GAGGATCTTGCCACTCTCCA -3´
Smarcd1	5´ -GACGATGACTGATGTGGTGGG-3´<br>5´ -ACCTTGGAGTAGAAGTATCGGC-3´
Smarce1	5´ -AAAAGACCATCTTATGCCCCAC-3´<br>5´ -CCTGTAGTTGTTGTAGGCGAG-3´
Vim	5’ –GTGCGCCAGCAGTATGAAAG-3’<br>5’ – CATCGTTGTTCCGGTTGG- 3’
